# Bridging Population Patterns and Individual Prediction: Framework for Prospective Multimorbidity Study

**DOI:** 10.2196/84261

**Published:** 2026-03-10

**Authors:** Qianyao Zhang, Runtong Zhang, Weiguang Ma, Butian Zhao, Xiaomin Zhu

**Affiliations:** 1 Department of Information Management, School of Economics and Management, Beijing Jiaotong University, No.3 Shangyuancun, Haidian District, Beijing, 100044, China, 86 010 51683854; 2 School of Management, Beijing University of Chinese Medicine, Beijing, China; 3 School of Mechanical, Electronic and Control Engineering, Beijing Jiaotong University, Beijing, China

**Keywords:** multimorbidity, latent transition analysis, LTA, deep learning, population-level patterns, personalized medicine

## Abstract

**Background:**

Multimorbidity has become a major global public health challenge. However, existing research primarily emphasizes the identification of disease patterns at the population level and lacks the capacity to provide predictive insights into individual future pattern membership. Bridging this gap is crucial for personalized prevention and management.

**Objective:**

This study aims to propose an innovative framework that integrates population-level multimorbidity pattern recognition with individual-level predictive modeling, thus advancing multimorbidity research from descriptive analysis to prospective multimorbidity pattern prediction.

**Methods:**

Using longitudinal health follow-up data, we first applied latent transition analysis (LTA) to identify temporally stable multimorbidity patterns. These patterns were subsequently transformed into predictive labels to construct a novel deep learning model, CLA-Net (Cross-Lag Attention Network). CLA-Net is designed to predict individual future multimorbidity patterns by leveraging the complementary strengths of Gated Recurrent Units (GRU) and transformer architectures. It introduces a bitemporal directed cross-attention mechanism to simultaneously capture temporal dependencies and complex feature interactions. We compared CLA-Net against several advanced baselines and conducted ablation studies to validate its architectural components.

**Results:**

In terms of pattern recognition, the LTA identified 5 clinically meaningful multimorbidity patterns: Cardiometabolic-Multisystem, Hypertension-Arthritis, Respiratory-Musculoskeletal, Metabolic Syndrome, and Gastritis-Arthritis. In terms of prediction, experimental results demonstrated that CLA-Net significantly outperformed all baseline models. CLA-Net achieved an accuracy of 0.8352 (SD 0.0048), a precision of 0.8326 (SD 0.0053), a recall of 0.8312 (SD 0.0056), and an *F*_1_-score of 0.8319 (SD 0.0051). Notably, it achieved an area under the curve of 0.9293, surpassing baseline models. Ablation studies confirmed the necessity of the dual-branch architecture and the directed cross-attention mechanism, as removing these components resulted in performance declines ranging from 0.93% to 2.50%.

**Conclusions:**

This study extends the scope of LTA beyond descriptive statistical modeling and establishes the scientific value of multimorbidity pattern prediction as an independent research task. By bridging population-level insights with individual-level prediction, the proposed framework provides a data-driven tool for the prospective prediction of future multimorbidity pattern membership conditional on survival, thereby supporting stratified disease management and care planning, rather than general risk stratification for acute or end-stage deterioration. This offers new methodological and practical value for precision medicine and public health policymaking.

## Introduction

Multimorbidity, commonly defined as the coexistence of 2 or more chronic conditions within a single individual [1], has emerged as one of the most pressing public health challenges of the 21st century amid the accelerating global trend of population aging [2]. Worldwide, approximately one-third of adults have 2 or more chronic diseases, and the proportion exceeds 50% among individuals aged 60 years and older [3]. Multimorbidity not only substantially increases health care resource consumption and imposes a heavy burden on health systems, but also severely compromises patients’ quality of life, leading to a markedly higher risk of functional disability and premature mortality, thereby greatly increasing the complexity of chronic disease management and clinical decision-making [4,5]. Previous studies have demonstrated that the co-occurrence of chronic diseases is not a random phenomenon but tends to manifest as distinct multimorbidity patterns, in which specific combinations of diseases frequently occur in particular populations [6,7]. This suggests the potential existence of shared pathophysiological mechanisms, risk factors, or lifestyle determinants underlying these patterns [8]. Therefore, systematically identifying, understanding, and predicting these multimorbidity patterns holds strategic importance for the early detection of high-risk individuals, the advancement of precision medicine, and the optimization of health care resource allocation [9,10].

Although various methods for identifying multimorbidity patterns have been proposed, such as factor analysis, cluster analysis, latent class analysis (LCA), and network analysis [11-15], they share significant limitations and fall short of meeting the clinical demand for personalized and prospective management. First, most approaches group populations primarily based on disease prevalence or epidemiological characteristics, assuming homogeneity within groups while overlooking the inherent heterogeneity among individuals [14,16]. Such population-level stratification can facilitate the identification of common multimorbidity patterns at a macrolevel but fails to uncover microlevel differences within patterns, which is particularly inadequate for supporting fine-grained individualized management in clinical practice. In reality, even among patients belonging to the same multimorbidity pattern, disease trajectories may differ substantially. For example, some patients experience slow progression and remain stable over a long period, whereas others may rapidly develop multisystem dysfunction [17]. Neglecting such heterogeneity may directly result in biased risk assessments and inefficient resource allocation, thereby severely constraining the practical implementation of precision medicine. In addition, existing studies have mainly focused on static multimorbidity pattern mining [16,18], and the methods used often struggle to maintain consistency of pattern meanings in longitudinal data. This instability not only reduces the comparability of research findings but also weakens the ability to capture disease progression, thereby limiting the clinical interpretability and practical value of the conclusions.

To address the above challenges, latent transition analysis (LTA), a longitudinal latent variable model, has been introduced into health research. LTA can identify stable latent health states across multiple time points and ensure their consistency and comparability over time [19]. Compared to other longitudinal modeling approaches, LTA offers distinct advantages for identifying multimorbidity evolution. Unlike growth mixed models or latent growth curve models, which primarily characterize continuous trajectories of a single variable (eg, functional decline over time), LTA is specifically designed to model transitions between discrete, qualitative latent statuses, aligning perfectly with the categorical nature of multimorbidity patterns. Furthermore, in contrast to standard Markov chain models that rely on observed states, LTA separates measurement error from true structural transitions by inferring latent classes from multiple observed indicators, thereby ensuring more robust and clinically meaningful pattern identification. By modeling follow-up data, LTA can effectively capture heterogeneity within populations and provide new perspectives for understanding the dynamic evolution of multimorbidity patterns [20,21]. However, LTA is essentially a descriptive statistical method, with its primary focus on identifying and summarizing patterns from historical or current data, while lacking the capacity to predict individual future health states. This limitation hinders its ability to meet urgent clinical needs, such as providing precise early warnings for high-risk individuals, developing timely intervention strategies, and guiding personalized treatment planning. Therefore, constructing predictive models that can accurately forecast individuals’ future multimorbidity patterns carries substantial clinical value and practical significance.

Predicting individual future multimorbidity patterns requires accounting for both the temporal evolution of health states and the complex interactions among multidimensional health features. On the one hand, the progression of chronic diseases exhibits marked and highly nonlinear temporal dependencies. A patient’s current health status not only reflects their immediate physiological condition but also embodies the evolution from the previous health state, reflecting the staged characteristics of disease progression [22]. Ignoring such sequential state transitions weakens a model’s ability to capture disease progression trends, thereby limiting its capacity to deliver reliable forward-looking predictions. On the other hand, multidimensional health features, including lifestyle factors, preexisting conditions, and functional indicators, are interconnected through intricate nonlinear couplings and synergistic effects. These dynamic interactions drive the formation and evolution of multimorbidity patterns [23]. Many conventional statistical methods, such as logistic regression, support vector machines, random forests, gradient boosting trees, and k-nearest neighbors (KNN), perform well in modeling static feature associations [24,25]. However, when confronted with high-dimensional, nonlinear, and temporally dependent medical data, their modeling capacity is limited, making it difficult to simultaneously capture nonlinear state transitions and deep couplings among features. Therefore, there is an urgent need to develop a model that overcomes these limitations by effectively integrating sequential health state changes and feature coupling effects in longitudinal data, thus enabling precise and prospective prediction of individual future multimorbidity patterns and providing robust data support for clinical decision-making.

Based on the above discussion, this study proposes an innovative framework that integrates population-level multimorbidity pattern recognition with precise prediction of individual future states, thereby advancing multimorbidity research from macrolevel description to prospective application. Specifically, the framework first uses LTA to identify population-level multimorbidity patterns with temporal consistency and clinical stability from longitudinal follow-up data, and then transforms these patterns into individual predictive labels. On this basis, we designed the Cross-Lag Attention Network (CLA-Net), which leverages immediate longitudinal context to model short-term, nonlinear state transitions and complex feature interactions among health variables, thereby enabling accurate prediction of individual future multimorbidity patterns.

The contributions of this study can be summarized in 3 aspects. First, we propose a novel cross-paradigm framework that bridges a statistical latent-variable modeling approach with deep representation learning for longitudinal multimorbidity research. Specifically, population-level multimorbidity patterns identified through LTA are leveraged as clinically interpretable supervisory signals for individual-level prediction. By integrating latent structure discovery with prospective modeling, this framework overcomes the limitation of traditional multimorbidity studies that are largely restricted to retrospective pattern characterization and establishes multimorbidity pattern prediction as a distinct and clinically meaningful research task.

Second, we propose a novel hybrid neural network architecture, CLA-Net, for multimorbidity pattern prediction. CLA-Net integrates the inherent strength of Gated Recurrent Units (GRU) in efficiently modeling short-term, nonlinear state transitions with the powerful representational capacity of the transformer architecture in capturing complex feature interactions. This design surpasses the capacity of traditional linear models, enabling deep modeling of the intricate couplings within medical data. Furthermore, CLA-Net incorporates a bitemporal directed cross-attention mechanism, which establishes directional information channels between the preceding and current health states. This mechanism facilitates feature extraction across time steps and dynamic association modeling, allowing the network to simultaneously perceive and effectively integrate dynamic state changes and feature coupling effects within complex longitudinal medical data.

Third, the experimental results demonstrate that the proposed CLA-Net model significantly outperforms other mainstream baseline models. Further ablation studies confirm that the synergistic integration of GRU and transformer, together with the bitemporal directed cross-attention mechanism, is the key factor driving the performance improvement. These findings not only provide strong evidence for the superiority of CLA-Net and the soundness of its design but also offer new insights and paradigms for model development in the field of multimorbidity pattern prediction.

## Methods

### Overview

**Figure 1.**
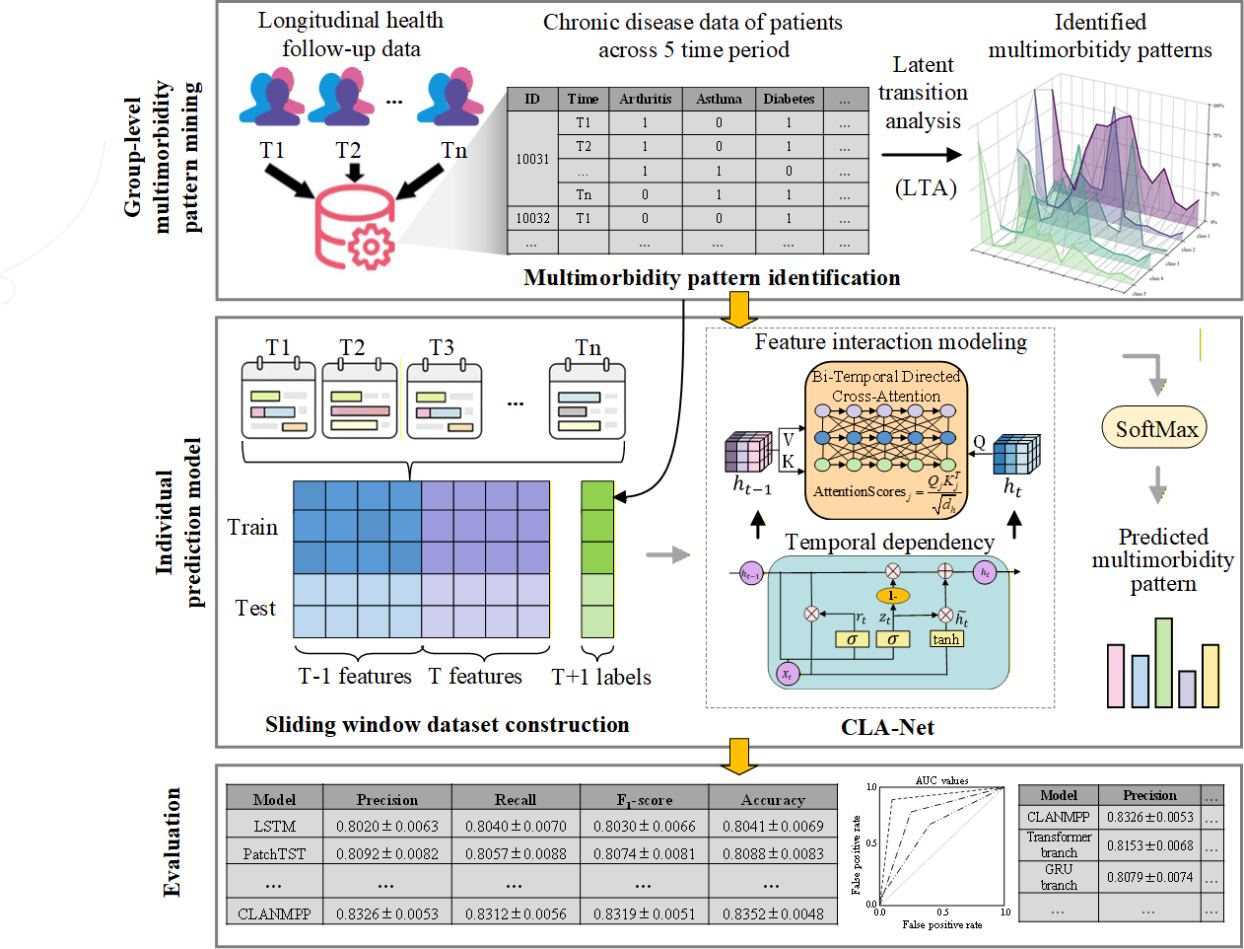
Proposed framework for multimorbidity pattern recognition and prediction. CLA-Net: Cross-Lag Attention Network; LTA: latent transition analysis.

We designed a novel research framework that integrates multimorbidity pattern recognition with individualized prediction. The overarching idea is to first identify population-level latent multimorbidity patterns with temporal consistency from longitudinal health data, and then build a deep learning model to predict each individual’s future pattern membership, thereby establishing a unified framework that bridges population-level recognition and individual-level prediction. As illustrated in Figure 1, the proposed framework consists of 3 stages. In the first stage, LTA is applied to longitudinal health records to identify latent multimorbidity patterns at the population level, ensuring that these patterns maintain consistent clinical meaning and comparability across different time points, thus providing a stable and reliable label basis for subsequent individual prediction tasks. In the second stage, based on the LTA-derived pattern labels, we design and construct the CLA-Net, which integrates temporal dependencies and complex feature interactions to enable accurate prediction of individual future pattern membership. In the third stage, we conduct a comprehensive performance evaluation and validation of the prediction model.

### Multimorbidity Pattern Identification

This study used SAS 9.4 (SAS Institute) to apply LTA for identifying the latent class structures of population-level multimorbidity patterns across 5 waves of China Health and Retirement Longitudinal Study (CHARLS) data. LTA is a longitudinal analytic method based on finite mixture models, which can detect unobserved health subgroups (latent statuses) within the sample across multiple time points. By imposing measurement invariance constraints, LTA ensures temporal consistency and stability of the latent classes [20], thereby guaranteeing that the identified classes maintain comparable and clinically meaningful interpretations across different time points. This provides stable and reliable classification labels for subsequent individual prediction modeling tasks. To control classification error and enhance model stability, we adopted a 2-step analytic strategy [26]. The detailed procedure is as follows: in step 1, we first conducted LCA separately at each time point to explore the optimal number of latent class partitions. In the model specification process, we considered different numbers of latent classes (ranging from 2 to 6) [26]. Model fit was evaluated using the Akaike Information Criterion (AIC), Bayesian Information Criterion (BIC), sample-size adjusted BIC (SaBIC), and entropy, to determine the optimal class solution.

Specifically, the AIC balances model fit and complexity, with smaller values indicating better fit [27]. The BIC adds a penalty for sample size, favoring more parsimonious models and thus being more conservative in class determination, particularly in large samples [28]. The SaBIC further adjusts BIC for sample size, offering greater robustness in large-sample contexts [29]. Entropy (ranging from 0 to 1) measures the certainty of individual classification, with values above 0.80 generally indicating high classification accuracy [30]. In addition, we used the Bootstrap Likelihood Ratio Test (BLRT) to compare log-likelihood differences between adjacent class models, in order to assess whether adding an extra class significantly improves model fit [31,32].

In step 2, after determining the optimal number of classes, we conducted chi-square tests on the log-likelihood values of each model [31] and performed invariance testing of class structures across different measurement time points, in order to verify whether the meanings of multimorbidity classes remained consistent over time [31]. This step is a critical prerequisite for ensuring that classes retain the same interpretive meaning across time points, thereby enhancing the interpretability and stability of the model [33].

In this study, LTA is used as a population-level structure discovery tool to identify clinically stable multimorbidity phenotypes, rather than as a component optimized for downstream predictive performance. The primary objective of LTA in our framework is to define a consistent and interpretable latent outcome space that reflects the underlying disease co-occurrence structure at the population level. Accordingly, LTA was estimated using the full longitudinal dataset across all available survey waves to maximize phenotype stability and clinical interpretability by leveraging the complete temporal information within the observed follow-up window. Within this 5-wave longitudinal design, the use of all waves enables the identified latent classes and transition patterns to reflect the evolution of multimorbidity structures across the entire observed period, rather than being driven by partial or wave-specific information. This design choice allows the latent class definitions to be less sensitive to sampling variability across individual waves and ensures that the resulting multimorbidity patterns are representative of the population dynamics observed within the study timeframe, rather than tailored to a specific subsample.

### Proposed CLA-Net Framework

#### Multimorbidity Pattern Prediction Dataset

This study constructed a task-oriented dataset for prospective prediction of multimorbidity patterns based on the CHARLS, a nationally representative longitudinal survey in China. To bridge the gap between population-level pattern discovery and individual-level prediction, we constructed a supervised learning task using LTA-derived latent classes as predictive targets. Since LTA is a probabilistic model, it outputs the posterior probability of an individual belonging to each latent class rather than a deterministic category. To transform these probabilistic outputs into deterministic prediction labels for CLA-Net, we used a maximum posterior probability assignment strategy. Specifically, for each individual *n* at time step *t*, the LTA model calculates a posterior probability vector ***P****_n,t_* = [*p*_1_, *p*_2_,…, *p_K_*], where *K* is the number of latent patterns. The ground truth label *y_n,t_* is assigned to the class with the highest probability:


(1) $$y_{n,t}=\arg\max_{k\in \left\{1,...,K\right\}}(P_{n,t}^{\left(k\right)})$$

These deterministic labels (*y_n,t_*) serve as the supervisory signals (ground truth) for training the CLA-Net.

We used 2 consecutive follow-up waves as the input window to predict the multimorbidity pattern at the subsequent wave. In CHARLS, the intervals between survey waves are 2‐3 years, which align with the slow evolution and long-term accumulation process of chronic diseases. Although the intervals vary slightly, the progression of chronic conditions is characterized by slow variables (ie, gradual progression, stable trends, and limited abrupt changes), making 2‐3 years sufficient to capture substantial changes in individual health status while reducing noise from short-term fluctuations. This temporal span is clinically reasonable for modeling chronic disease progression. According to the study objectives, we reorganized the raw data using a sliding-window approach to construct 3 temporally progressive subdatasets, each corresponding to a “past–current → future” window structure:

- Subdataset 1: 2011 features as early input (**x***_t–1_*), 2013 features as current input (**x***_t_*), and 2015 LTA-derived multimorbidity patterns as prediction labels (*y_t+1_*)
- Subdataset 2: 2013 features as (**x***_t–1_*), 2015 features as (**x***_t_*), and 2018 multimorbidity patterns as (*y_t+1_*)
- Subdataset 3: 2015 features as (**x***_t–1_*), 2018 features as (**x***_t_*), and 2020 multimorbidity patterns as (*y_t+1_*)

The 3 subdatasets were concatenated by rows to form a unified prediction dataset, structured as triplets (**x***_t–1,_* **x***_t,_ y_t+1_*), where **x***_t–1_* and **x***_t_* represent feature vectors from 2 consecutive time points, and *y_t+1_* denotes the LTA-identified future multimorbidity pattern label. This construction preserves the natural temporal evolution of disease states without relying on long-sequence assumptions, while effectively augmenting the training data. It enables the model to learn multistage health state transitions within the same population, thereby providing a robust data foundation for subsequent individual-level prospective prediction.

#### Overview of the Model Architecture

**Figure 2.**
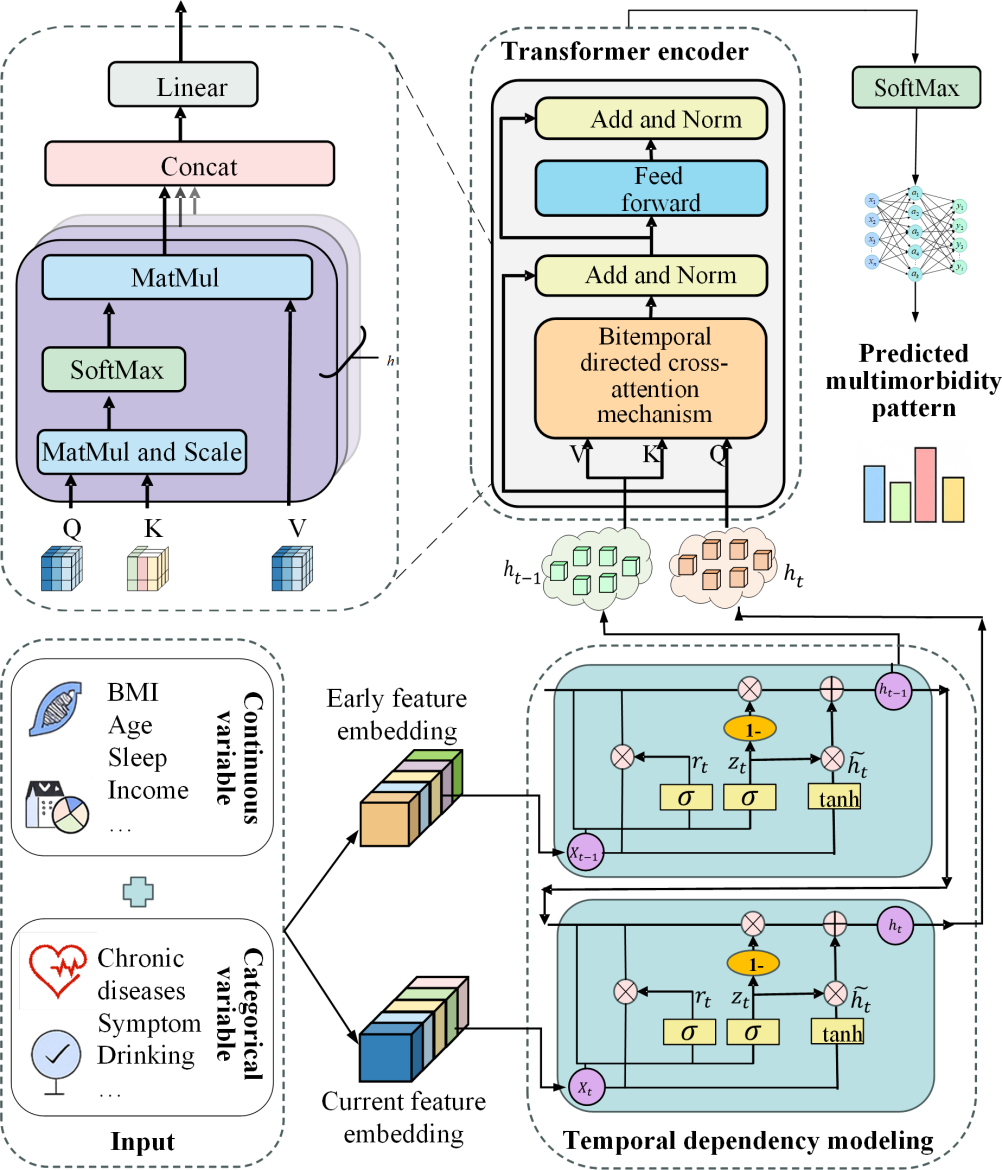
The architecture of the Cross-Lag Attention Network.

This study proposes an innovative deep learning model, named the CLA-Net. CLA-Net integrates the sequential state encoding capability of GRU with the global feature interaction advantages of an improved transformer architecture, enabling fine-grained cross-time feature interactions through a bitemporal directed cross-attention mechanism. The model is composed of 4 core modules: an input embedding layer, a temporal GRU encoder, a transformer encoding layer based on the bitemporal directed cross-attention mechanism, and a classifier layer. An overview of the model architecture is shown in Figure 2.

#### Components of CLA-Net

##### Feature Embedding Layer

The input features include continuous variables (eg, biomarkers and cognitive scores) and binary indicator variables (eg, disease diagnoses and medication use). Socioeconomic variables, including total household income and per capita household consumption, were inspected for extreme values prior to normalization. Given the heavy-tailed distributions of these variables, a percentile-based outlier handling strategy was applied to mitigate the undue influence of extreme observations while preserving the original scale of the data. Min-max normalization was then used to rescale these variables to a common range, ensuring numerical stability and consistency with the scaling of other continuous inputs used in the model. Binary features were kept unchanged. Subsequently, heterogeneous features are mapped into a unified representation space through learnable linear transformations:


(2) $$X_{t-1}W_{e}x_{t-1}+b_{e}\in R^{d}$$

(3) $$X_{t}=W_{e}X_{t}+b_{e}\in R^{d}$$

where $x_{t}\in R^{D}$ denotes the raw input features, $X_{t}\in R^{d}$denotes the embedded representation, $W_{e}\in R^{d\times D}$and $b_{e}\in R^{d}$are shared parameters, *D* denotes the input dimension, and *d* represents the hidden space dimension.

##### Temporal GRU Encoder

After feature embedding, the representations of features from the earlier time point (**X***_t–1_*) and the current time point (**X***_t_*) are concatenated into a 2-step sequence, which is then fed into a GRU for temporal modeling. Through the update mechanism of its hidden states, the GRU efficiently captures the sequential evolution of the disease process, ensuring that the nonlinear dynamics of health state transitions are explicitly modeled. This design enables the network to capture the critical short-term progression from *t*–1 to *t* and to explicitly model the nonlinear dynamics underlying near-term health state transitions.


(4) $$h_{t-1}=GRU(X_{t-1},h_{init})$$

(5) $$h_{t}=GRU(X_{t},h_{t-1})$$

where **h***_init_* denotes the initial hidden state, and **h***_t–1_* and **h***_t_* represent the hidden state outputs at the 2 time steps. The internal computational mechanism of the GRU is defined as follows:


(6) $$r_{t}=\sigma \left(W_{r}X_{t}+U_{r}h_{t-1}+b_{r}\right)$$

(7) $$z_{t}=\sigma \left(W_{z}X_{t}+U_{z}h_{t-1}+b_{z}\right)$$

(8) $${\tilde{h}}_{t}=\tanh\left(W_{h}X_{t}+U_{h}\left(r_{t}⊙h_{t-1}\right)+b_{h}\right)$$

(9) $$h_{t}=(1-z_{t})⊙h_{t-1}+z_{t}⊙{\tilde{h}}_{t}$$

Here, **X***_t_* denotes the input at time step *t*. **r***_t_* is the reset gate, which controls how much information from the previous time step is retained. **z***_t_* is the update gate, which determines the proportion of information to be updated at the current time step. $\tilde{h_{t}}$ represents the candidate’s hidden state. σis the sigmoid activation function, and ⊙ denotes elementwise multiplication. W_*_ and U_*_ are learnable weight matrices, and b_*_ is the bias vector.

##### Transformer Encoding Layer

###### Bitemporal Directed Cross-Attention Mechanism

Although GRUs are effective at encoding immediate longitudinal context via gated state updates, they have limited capacity to explicitly characterize fine-grained interactions among heterogeneous features, especially when the predictive signal arises from short-term, nonlinear state transitions rather than long-range history. In our setting, an individual’s future multimorbidity pattern is shaped not only by the recent state representation but also by complex cross-feature influences that operate across adjacent time points. To address this, we adopt an asymmetric attention paradigm, in which features from the current time point are used as the query vector **Q**, while features from the earlier time point serve as the keys (**K**) and values (**V**) in the attention computation, enabling the model to attend to historical representations when updating the current state. This design reflects the directional interactions between features across time. For each attention head *j* ∈ {1,...,*H*}, the GRU outputs are first projected into the **Q**, **K**, and **V** spaces:


(10) $$\left\{ \begin{array}{l} Q_{j}=h_{t}W_{Q}^{\left(j\right)}\\ K_{j}=h_{t-1}W_{K}^{\left(j\right)}\\ V_{j}=h_{t-1}W_{V}^{\left(j\right)} \end{array}\right.$$

where *Q*_*j*_, *K*_*j*_, and *V*_*j*_ denote the query, key, and value representations of the *j*th attention head, which are vector-valued representations derived from the input hidden states. $W_{Q}^{\left(j\right)},\;W_{K}^{\left(j\right)},\;W_{V}^{\left(j\right)}\in R^{d\times d_{h}}$ denotes the projection matrix of the *j*th head, *d* is the input dimension, and *d*_h_=*d/H* is the dimension of each attention head. The attention scores are computed using scaled dot-product attention:


(11) $${Attention\;scores}_{j}=\frac{Q_{j}K_{j}^{T}}{\sqrt{d_{h}}}$$

The scaling factor $1/\sqrt{d_{h}}$ is critical for maintaining gradient stability. Without scaling, the dot-product values increase with feature dimensionality, which may push the SoftMax function into regions with extremely small gradients. The computation for each attention head is defined as


(12) $${head}_{j}=Attention(Q_{j},K_{j},V_{j})=SoftMax\;\left(\frac{Q_{j}K_{j}^{T}}{\sqrt{d_{h}}}\right)V_{j}$$

After concatenating the outputs of all attention heads, a linear transformation is applied to obtain the multi-head cross-attention output:


(13) $$\text{MultiHeadCrossAttn}(h_{t-1},h_{t})=\text{Concat}({head}_{1},\ldots ,{head}_{H})\;W_{o}$$

where **W***_O_* denotes the output projection matrix. Through the multihead mechanism, the model can learn distinct attention patterns in different representation subspaces, allowing it to attend to diverse types of feature relationships simultaneously and thereby enhancing its expressive capacity.

###### Stacked Transformer Encoder Architecture

To capture the hierarchical patterns of disease progression, we use a stack of *L* transformer encoder layers. Each layer builds upon the representations from the previous one, thereby progressively modeling more complex temporal dependencies. Each layer, *l* ∈ {1,..,*L*} consists of 2 sublayers with residual connections. Specifically, Equation 14 corresponds to the bitemporal directed cross-attention mechanism sublayer, while Equation 15 represents the positionwise feed-forward network (FFN) sublayer:


(14) $${\hat{z}}^{(l{)}^{\prime }}=\text{LayerNorm}\;\left(z^{\left(l-1\right)}+\text{Dropout}\;\left(\text{MultiHeadCrossAttn}(h_{t-1},\;z^{\left(l-1\right)})\right)\right)$$

(15) $$z^{\left(l\right)}=LayerNorm\;\left({\hat{z}}^{\left(l\right)}+Dropout(FFN({\hat{z}}^{\left(l\right)}))\right)$$

Here, the positionwise FFN uses the ReLU activation function and incorporates dropout to prevent overfitting, defined as


(16) $$FFN(x)=ReLU(xW_{1}+b_{1})W_{2}+b_{2}$$

where **W**_1_ is the weight matrix of the first feed-forward layer, which expands the feature dimension by a factor of 4 to enhance nonlinear representational capacity. **W**_2_ is the weight matrix of the second feed-forward layer, which projects the expanded dimension back to the original size *d*. **b**_1_ and **b**_2_ denote the bias vectors for the first and second layers, respectively. After passing through *L* stacked layers, we obtain the final fused representation **z**^(^*^L^*^)^, which simultaneously encodes early and later temporal information of patients and is further enriched through direct cross-time feature interactions.

### Classifier Layer

The fused features **z**^(^*^L^*^)^ output by the transformer encoder are fed into a 2-layer fully connected neural network as the classifier. This classifier consists of a linear layer, a ReLU activation function, a dropout layer, and a final linear output layer, which maps the fused features into probability distributions over multimorbidity patterns.

#### First Layer: Linear Mapping and Nonlinear Activation

The first layer of the classifier consists of a linear mapping followed by a nonlinear activation function. This step is crucial for transforming the fused features into a more complex representation. The output of this layer is computed as follows:


(17) $$o=ReLU\;\left(z^{\left(L\right)}W_{1}^{\left(c\right)}+b_{1}^{\left(c\right)}\right)$$

where $W_{1}^{\left(c\right)}\in R^{d\times d}$ and $b_{1}^{\left(c\right)}\in R^{d}$ are the weight matrix and bias vector of the first layer, respectively, and ReLU denotes the activation function. A dropout regularization is applied after this layer to reduce the risk of overfitting.

#### Second Layer: Output Mapping

The second layer of the classifier maps the transformed features to output logits, representing the class scores. This layer computes the logits as follows:


(18) $$y_{\text{logits}}=o\;W_{2}^{\left(c\right)}+b_{2}^{\left(c\right)}$$

where $W_{2}^{\left(c\right)}\in R^{d\times C}$ and $b_{2}^{\left(c\right)}\in R^{C}$ represent the weight matrix and bias vector of the second layer, respectively, and *C* denotes the number of output classes. The final prediction **y**_logits_ is obtained by applying the SoftMax function to transform the outputs into probabilities:


(19) $$\hat{y}=SoftMax(y_{\text{logits}})=\frac{\exp(y_{\text{logits},\;c})}{\sum_{c^{\prime }=1}^{C}\exp(y_{\text{logits},\;c^{\prime }})}$$

### Loss Function

We trained the model using the standard cross-entropy loss function for multiclass, single-label classification. Based on the predicted probability distribution $\hat{y}$ output by the classifier, the cross-entropy loss is defined as


(20) $$L=-\frac{1}{N}\sum_{i=1}^{N}\sum_{c=1}^{C}y_{i}^{\left(c\right)}\log\;\left({\hat{y}}_{i}^{\left(c\right)}\right)$$

where *C* denotes the number of classes, $y_{i}^{\left(c\right)}$is the one-hot encoded true label of the *i*th sample for class *c*, and $y_{i}^{\left(c\right)}$represents the predicted probability that the *i*th sample belongs to class *c*. The objective is to minimize the average loss across all training samples. Cross-entropy is particularly suitable for our multiclass single-label task because it encourages the network to assign high probability to the correct class while penalizing confident but incorrect predictions. Since the dataset has a relatively balanced class distribution, no class weighting was applied in the loss function. The use of SoftMax outputs with cross-entropy is equivalent to maximizing the likelihood of the correct class and is a standard choice for neural network classifiers.

Algorithm 1 in Textbox 1 presents the pseudocode of the CLA-Net model. The algorithm details how each component is sequentially executed on each batch of data.

Textbox 1. **Algorithm 1: CLA-Net (Cross-Lag Attention Network) model.**

**Input**:

$x_{t}$: Features at time *t* (continuous features $x_{t}^{cont}$ , discrete features $x_{t}^{disc}$)

*x_t_*_–1_: Features at time t–1 (continuous features $x_{t-1}^{cont}$ , discrete features $x_{t-1}^{disc}$)

y: Label (5-class classification)

**Output**: Predicted probability distribution **p**, cross-entropy loss *L*

Process:

1: // *1. Feature preprocessing and embedding*

2: $e_{t-1}\leftarrow Embedding\;\left(x_{t-1}^{disc}\right)$ *// Embedding of discrete features*

3: $e_{t}\leftarrow Embedding\;\left(x_{t}^{disc}\right)$

4: $c_{t-1}\leftarrow LayerNorm\;\left(x_{t-1}^{cont}\right)$ *// Normalization of continuous features*

5: $c_{t}\leftarrow LayerNorm\;\left(x_{t}^{cont}\right)$

6:$x_{t-1}\leftarrow Linear\;\left(Concat(e_{t-1},\;c_{t-1})\right)$ *// Concatenation of features at time t−1*

7: $x_{t}\leftarrow Linear\;\left(Concat(e_{t},\;c_{t})\right)$ *// Concatenation of features at time t*

8: $X_{t-1}\leftarrow W_{e}\;x_{t-1}+b_{e}$

9: $X_{t}\leftarrow W_{e}\;x_{t}+b_{e}$

10: *// 2. GRU-based temporal encoding*

11:$h_{t-1}\leftarrow GRU\;\left(X_{t-1},\;h_{init}\right)$ *// Hidden state at time t−1*

12: $h_{t}\leftarrow GRU\;\left(X_{t},\;h_{t-1}\right)$ *// Hidden state at time t*

13: *// 3. Transformer encoder based on the bitemporal directed cross-attention mechanism*

14: for do

15: for head $i=1,\ldots ,H_{a}$ do

16: $Q^{\left(i\right)}\leftarrow W_{Q}^{\left(i\right)}h_{t},\;\;K^{\left(i\right)}\leftarrow W_{K}^{\left(i\right)}h_{t-1},\;\;V^{\left(i\right)}\leftarrow W_{V}^{\left(head\right)}h_{t-1}$

17: $A^{\left(i\right)}\leftarrow Softmax\;\left(\frac{Q^{\left(i\right)}(K^{\left(i\right)}{)}^{⊤}}{\sqrt{d_{k}}}\right)$ *// Attention weights*

18:${head}^{\left(i\right)}\leftarrow A^{\left(i\right)}V^{\left(i\right)}$ *// Context vector*

19: end for

20: $Z\leftarrow Concat\;\left({head}^{\left(1\right)},\ldots ,{head}^{\left(H\right)}\right)W_{o}$

21: $U\leftarrow LayerNorm\;\left(h_{t}+Dropout(Z)\right)$ *// Residual connection and normalization*

22: $h_{t}\leftarrow LayerNorm\;\left(U+Dropout\;\left(FFN(U)\right)\right)$

23: end for

24: $\text{Let }z\leftarrow h_{t}$*// final fused representation*

25: *// 4. Classification and prediction*

26: o←Flatten(z)

27: $p\leftarrow Softmax\;\left(W_{f_{c}}\;o+b_{f_{c}}\right)$*// Output probability distribution*

28: $L\leftarrow -\sum_{i=1}^{5}y_{i}\log p_{i}$ *// Cross-entropy loss*

29: return **p**, *L*

### Training Configuration and Implementation Details

All experiments were conducted using PyTorch v1.7.0 (Facebook AI Research, Meta Platforms) and Python 3.8 (Python Software Foundation) on Ubuntu 18.04 (Canonical Ltd), with an NVIDIA RTX 4090 GPU (24 GB memory) and 32 GB RAM (CUDA 11.0). The proposed model and all baselines were trained under identical configurations and hyperparameters (Table 1). The main settings were 60 training epochs, mini-batch size of 64, embedding and GRU hidden dimensions set to 128, Adam optimizer with a learning rate of 0.0001 (determined via grid search), cross-entropy loss, and a dropout rate of 0.3 applied to both transformer layers and the classifier. Model weights were initialized using PyTorch defaults. During data preprocessing, feature normalization was fitted only on the training set and then applied to the validation and test sets. The dataset was partitioned into 80% training and 20% testing sets using a subject-wise splitting strategy based on unique individual identifiers. This approach ensures that all longitudinal observations from the same participant were assigned exclusively to a single subset, thereby preventing potential data leakage. From the training set, 20% (n/N) of the individuals were further held out as a validation set. During training, the macro-*F*_1_-score on the validation set was used as the criterion for early stopping and model selection. Only the best-performing model on the validation set was retained, and a final evaluation was conducted on the independent test set.

**Table 1.** Training hyperparameters.

Parameters or hyperparameters	Value
Epoch	60
Learning rate	0.0001
Dropout	0.3
Random seed	42
Batch size	64
Hidden dimension	128

### Evaluation Metrics

To evaluate the performance of the model in the multiclass classification task, we used commonly used metrics, including accuracy, precision, recall, and *F*_1_-score, and reported their macroaveraged values. Macroaveraging assigns equal weight to each class, thereby avoiding the bias of microaveraging, which tends to favor majority classes in imbalanced datasets. This provides a more comprehensive and balanced assessment of the model’s ability to identify all 5 multimorbidity patterns. The formulas for these evaluation metrics are as follows:


(21) $$Accuracy=\frac{\sum_{c=1}^{C}TP_{c}}{\sum_{c=1}^{C}\left(TP_{c}+FP_{c}+FN_{c}+TN_{c}\right)}$$

(22) $${Precision}_{c}=\frac{TP_{c}}{TP_{c}+FP_{c}}$$

(23) $${Recall}_{c}=\frac{TP_{c}}{TP_{c}+FN_{c}}$$

(24) $$F_{1}=2\times \frac{{Precision}_{c}\times {Recall}_{c}}{{Precision}_{c}+{Recall}_{c}}$$

where *TP_c_*, *FP_c_*, *FN_c_*, and *TN_c_* denote the true positives, false positives, false negatives, and true negatives for class *c*, respectively. Accuracy represents the proportion of correctly predicted samples to the total number of samples. Precision measures the proportion of samples predicted as a given class that actually belong to that class. Recall indicates the proportion of samples that are correctly identified among those that truly belong to a given class. The *F*_1_-score, defined as the harmonic mean of precision and recall, provides a balanced measure of the model’s classification performance for a given class. Macroaveraging is obtained by taking the arithmetic mean of the metric values across all classes. The formulas are defined as follows:


(25) $$\left\{ \begin{array}{rl} Macro\;-\;Precision & =\frac{1}{C}\sum_{c=1}^{C}{Precision}_{c},\\ Macro\;-\;Recall & =\frac{1}{C}\sum_{c=1}^{C}{Recall}_{c},\\ Macro\;-\;F_{1} & =\frac{1}{C}\sum_{c=1}^{C}{F_{1}}_{c} \end{array}\right.$$

In addition, we further introduced the receiver operating characteristic (ROC) curve and the area under the curve (AUC) as complementary evaluation metrics. While accuracy focuses on the correctness of class predictions, AUC assesses the ranking quality of predicted probabilities. Particularly in multiclass problems, a higher macroaveraged AUC together with higher accuracy provides stronger evidence that the model can achieve good discrimination across all classes, rather than merely benefiting from class size distributions. The ROC curve is plotted with the false positive rate (FPR) on the *x*-axis and the true positive rate (TPR) on the *y*-axis and is mathematically defined as:


(26) $$FPR=\frac{FP}{FP+TN},\;TPR=\frac{TP}{TP+FN}$$

AUC measures the overall discriminative ability of a model, with values ranging from 0 to 1, where values closer to 1 indicate stronger separation between positive and negative classes. Commonly, AUC >0.9 is considered excellent, 0.8‐0.9 good, 0.7‐0.8 acceptable, and <0.7 poor. For multiclass prediction, we adopted a one-vs-rest strategy, treating each class as positive against all others to compute class-specific ROC curves and AUC values. To validate the effectiveness of the proposed model, we compared it with multiple baselines under identical test conditions and reported AUC as a key performance metric. The evaluation procedures followed recommended practices for multiclass classification to ensure reliable assessment of predictive performance.

In addition to AUC-ROC, we also reported AU-PRC (area under the precision-recall curve), which provides a complementary evaluation of model performance by emphasizing the trade-off between precision and recall. AU-PRC is particularly informative in settings with class imbalance, as it focuses on the model’s ability to correctly identify positive instances without being overly influenced by true negatives. Similar to the ROC-based evaluation, AU-PRC was computed using a one-vs-rest strategy for multiclass prediction, and class-specific precision-recall (PR) curves were aggregated to assess overall performance.

### Baseline Models

#### Logistic Regression

A linear classification model that estimates class probabilities through a logistic function, serving as a simple and interpretable baseline to assess the benefit of nonlinear feature interactions [34].

#### Support Vector Machine

A margin-based classifier that constructs an optimal separating hyperplane in the feature space, enabling the evaluation of nonlinear decision boundaries through kernel-based learning [34].

#### Random Forest

An ensemble learning method that aggregates multiple decision trees trained on bootstrapped samples, capturing nonlinear feature relationships and improving robustness against overfitting [35].

#### XGBoost

A gradient boosting framework that builds additive tree-based models in a stagewise manner, designed to optimize predictive performance by modeling complex nonlinear interactions among features [36].

#### Convolutional Neural Network

Extracts local temporal features via convolutional kernels without explicitly modeling temporal order, serving to test the importance of temporal structure [37].

#### Long Short-Term Memory

Captures long-term dependencies through gating mechanisms, used to compare different recurrent units for short-sequence modeling [38].

#### Transformer

Models relationships across time steps using self-attention, with concatenated features fed into a standard transformer encoder [39].

#### PatchTST

Reduces computational complexity through a “patching” strategy, improving efficiency for long-sequence prediction [40].

#### iTransformer

Applies attention along the feature dimension rather than the temporal dimension, better capturing inter-variable relationships in multivariate time series [41].

#### Mamba

Mamba uses a selective state space mechanism to model long-term dependencies with linear complexity [42].

#### MambaTS

An enhanced variant of Mamba that incorporates improved state space mechanisms to further boost long-sequence prediction performance [43].

#### Long Short-Term Memory+Transformer

A variant of CLA-Net that replaces the GRU encoder with a long short-term memory (LSTM), used to compare the effects of different recurrent units.

To ensure a fair comparison with the deep learning models, the input configuration for all traditional machine learning baselines (logistic regression, support vector machine, random forest, and XGBoost) was aligned with the dual-time-point setup of CLA-Net. Specifically, the feature vectors from the historical time point ($X_{t-1}$) and the current time point ($X_{t}$) were concatenated to form a single flattened input vector [$X_{t-1}$, $X_{t}$]. This ensures that all models use the exact same longitudinal information, preventing any performance bias due to information asymmetry.

### Ethical Considerations

Ethical approval for the original data collection in the CHARLS project was granted by the Biomedical Ethics Review Committee of Peking University (approval number: IRB00001052-11015). All participants provided written informed consent at the time of enrollment. As this study involves a secondary analysis of publicly available, deidentified data, the requirement for additional informed consent was waived. To ensure privacy and confidentiality, all direct identifiers were removed from the dataset by the CHARLS team prior to public release. Furthermore, this paper and its supplementary materials do not contain any images or information that could identify individual participants.

## Results

### Dataset

**Figure 3.**
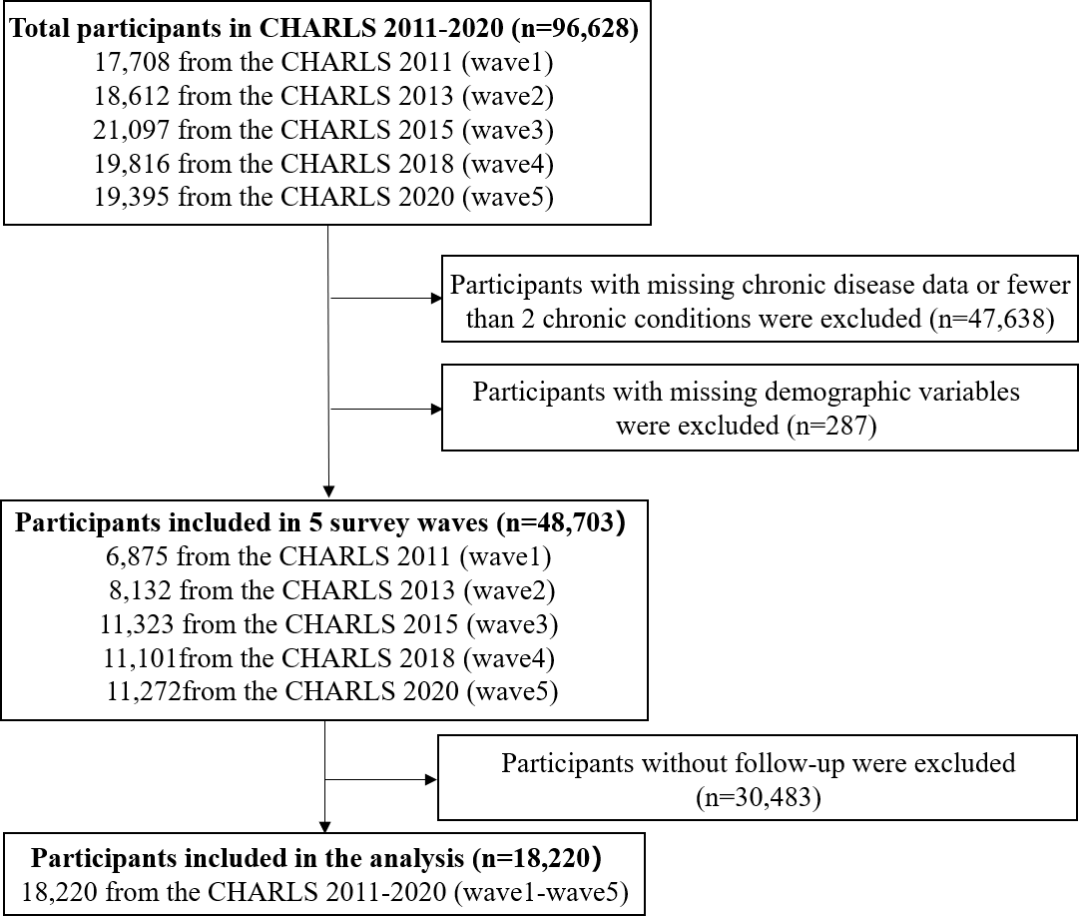
Participant selection flowchart. CHARLS: China Health and Retirement Longitudinal Study.

This study adopted a longitudinal cohort design using publicly available data from the CHARLS. CHARLS is a nationally representative longitudinal survey covering 28 provinces, 150 counties and districts, and 450 communities, and provides extensive demographic, chronic disease, and other health-related information. Using 2011 as the baseline, we included respondents with at least 2 chronic conditions and complete demographic data who participated in all 5 waves, yielding 3644 individuals and 18,220 person-wave observations. The sample selection process is shown in Figure 3. Attrition during follow-up occurred due to both mortality and nonmortality loss to follow-up. Specifically, the without-follow-up observations (30,483 person-wave observations) corresponded to 1617 person-wave observations attributable to death and 28,866 person-wave observations attributable to other reasons for nonparticipation.

Data preprocessing included logical error correction and outlier removal. Regarding missing value treatment, to strictly prevent data leakage, we adhered to a rigorous “split-then-impute” strategy. The dataset was first partitioned into training and independent test sets. Subsequently, missing values were handled using the MiceForest method for multiple imputation [44]. Crucially, the imputation model was fitted exclusively on the training set. The MiceForest method is based on random forests and predictive mean matching, allowing it to capture nonlinear relationships and improve imputation accuracy [45,46]. To ensure data quality, variables with more than 50% missingness were excluded, and only those with relatively low missingness were retained [45].

The final dataset variables are summarized in Table S1 in Multimedia Appendix 1 and include (1) individual identifiers and survey time (ID and year); (2) 14 chronic disease variables used for LTA-based identification and transition modeling of multimorbidity patterns, which also served as target variables in the prediction task; and (3) symptom variables along with demographic, behavioral, psychological, socioeconomic, and macropolicy features, which were used as input features for the prediction model. A final cohort of 3644 individuals with multimorbidity who maintained participation across all 5 survey waves was included in the analysis. Table 2 presents the baseline sociodemographic characteristics of the participants (recorded in 2011). The cohort was predominantly female (n=2132, 58.51%) and comprised mainly middle-aged and older adults, with 93.33% (n=3401) of participants aged between 45 and 74 years. In terms of socioeconomic status, the sample was characterized by relatively low educational attainment, as 90.12% (n=3284) of participants had an education level below junior high school. The majority were married (n=3283, 90.09%) and resided in rural areas (n=2306, 63.28%), while less than half (n=1550, 42.54%) were covered by pension insurance.

**Table 2.** Demographic characteristics.

Variables	Values
Gender, n (%)	
Men	1512 (41.49)
Women	2132 (58.51)
Age (years), n (%)	
<18	1 (0.03)
18‐44	65 (1.78)
45‐59	1881 (51.62)
60‐74	1520 (41.71)
≥75	177 (4.86）
Education level, n (%)	
Below junior high school	3284 (90.12)
High school or vocational training	313 (8.59)
Higher education	47 (1.29)
Marital status, n (%)	
Unmarried	361 (9.91)
Married	3283 (90.09)
Residence, n (%)	
Rural	2306 (63.28)
Urban	1338 (36.72)
Pension coverage, n (%)	
No	2094 (57.46)
Yes	1550 (42.54)

### LTA-Based Identification of Multimorbidity Patterns

To clarify the consistency and heterogeneity of multimorbidity pattern structures across different time points, we first conducted LCA at each wave to provide the basis for subsequent LTA. The model fit results (Table 3) indicated that the 5-class solution performed best across multiple criteria: at T1, T2, T3, and T5, it achieved the lowest AIC and SaBIC values with good classification quality (entropy=0.838‐0.926); at T4, although the 4-class and 5-class solutions showed comparable entropy, the 5-class model outperformed in AIC, BIC, and SaBIC. The BLRT consistently supported the 5-class solution across all time points (all *P*<.001). Moreover, the class distributions of the 5-class solution were relatively stable and clinically plausible across waves. Taken together, the 5-class model was selected as the optimal solution for the LTA.

After establishing the 5-class structure at each time point, we tested measurement invariance to ensure the validity of cross-time comparisons. As shown in Table 4, constraining item-response probabilities to be equal across time did not significantly worsen model fit compared to the freely estimated model (∆*χ*²_75_=88.7; *P*=.14). This nonsignificant likelihood ratio test confirmed measurement invariance, indicating that the latent classes maintained consistent meanings across all 5 waves.

**Table 3.** Latent class analysis results for multimorbidity patterns at 5 time points based on the CHARLS (China Health and Retirement Longitudinal Study) database.

Time and class		Percent (%)	AIC^a^	BIC^b^	SaBIC^c^	Entropy	BLRT^d^
T1							
	2	40.5/59.5	4196.03	4375.85	4283.70	0.720	—^e^
	3	47.8/16.5/35.8	3598.93	3871.77	3731.96	0.802	<0.001
	4	21.4/15.6/34.8/28.2	3378.95	3744.80	3557.32	0.839	<0.001
	5	11.6/15.3/39.8/9.4/23.9	3230.96	3782.83	3500.03	0.926	<0.001
	6	17.1/15.0/32.5/19.4/14.4/1.6	3293.62	3752.49	3517.35	0.856	<0.001
T2							
	2	37.7/62.3	4083.43	4263.25	4171.11	0.711	—
	3	46.0/15.3/38.6	3498.78	3771.62	3631.81	0.803	<0.001
	4	10.7/15.1/40.3/33.8	3285.65	3651.50	3464.03	0.860	<0.001
	5	11.1/13.6/39.5/2.1/33.7	3197.59	3656.45	3421.32	0.889	<0.001
	6	14.7/12.8/34.9/7.4/27.6/2.6	3167.49	3719.36	3436.56	0.878	<0.001
T3							
	2	36.6/63.4	4199.14	4378.97	4286.82	0.701	—
	3	39.5/12.7/47.8	3603.58	3876.42	3736.61	0.800	<0.001
	4	20.5/12.0/35.6/32.0	3446.95	3812.80	3625.33	0.802	<0.001
	5	5.0/9.6/33.0/24.1/28.3	3238.82	3697.68	3462.54	0.838	<0.001
	6	24.8/10.2/30.1/16.8/13.6/4.6	3218.26	3770.13	3487.33	0.884	<0.001
T4							
	2	41.3/58.7	5174.42	5354.24	5262.09	0.687	—
	3	18.4/42.2/39.4	4448.84	4721.68	4581.87	0.798	<0.001
	4	11.2/44.0/35.8/9.0	4034.00	4399.85	4212.37	0.849	<0.001
	5	8.7/9.8/29.9/26.8/24.7	3869.07	4327.93	4092.79	0.842	<0.001
	6	8.0/9.9/30.8/19.4/27.6/4.2	3832.77	4384.64	4101.84	0.834	<0.001
T5							
	2	49.0/51.0	5576.80	5756.62	5664.47	0.694	—
	3	22.7/35.5/41.8	4771.20	5044.04	4904.23	0.812	<0.001
	4	11.8/34.9/43.4/9.9	4272.68	4638.53	4451.05	0.848	<0.001
	5	9.8/12.5/32.2/37.2/8.2	4087.68	4639.55	4356.75	0.849	<0.001
	6	13.2/9.0/30.6/34.8/8.8/3.6	4135.51	4594.37	4359.23	0.839	<0.001

a AIC: Akaike Information Criterion.

b BIC: Bayesian Information Criterion.

c SaBIC: sample-size adjusted Bayesian Information Criterion.

d BLRT: Bootstrap Likelihood Ratio Test.

e Not applicable.

**Table 4.** Likelihood ratio tests for measurement invariance of the latent transition analysis model.

Model	LogLik^a^	−2LL^b^	Chi-square^c^ (*df*)^d^	*P* value
M_free^e^	−97100	194,200	—^f^	—
M_invariant^g^	−97144.4	194,288.7	88.7 (75)	.14

a LogLik: log-likelihood.

b −2LL: −2 log-likelihood.

c Δ*χ*²: chi-square difference test.

d Δdf: difference in degrees of freedom.

e M_free: model with all parameters freely estimated across time.

f Not applicable.

g M_invariant: model with item-response probabilities constrained to be equal across time points.

**Figure 4.**
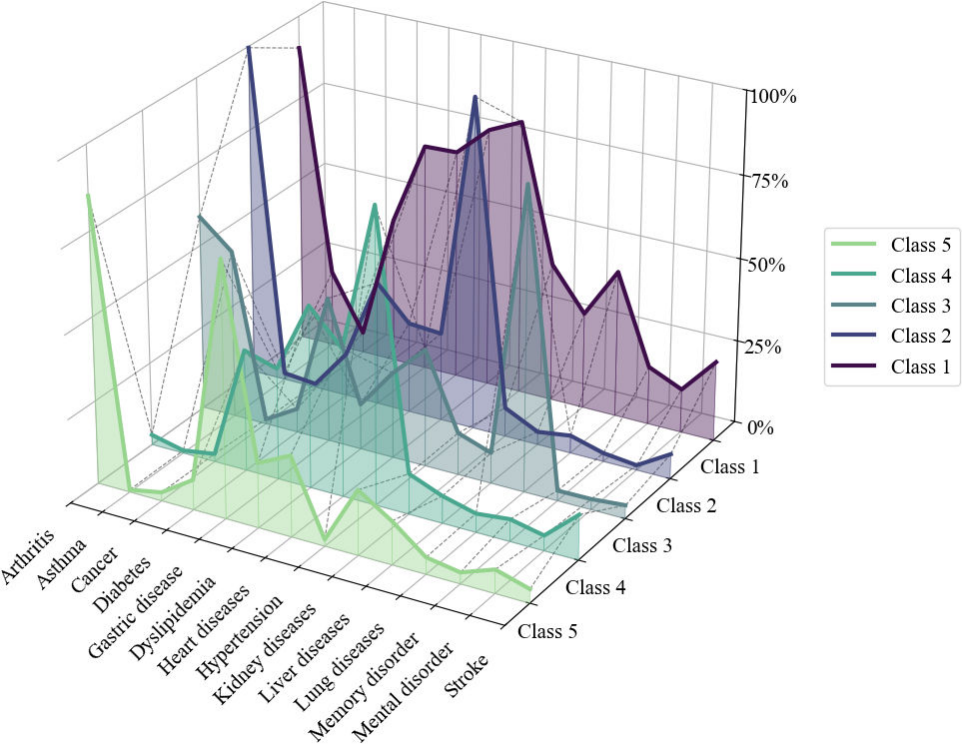
Disease probabilities in the 5 multimorbidity patterns.

The LTA clustering results for the 5-class model are shown in Figure 4. Class 1 (19.8%) had the highest overall disease burden, with nearly universal hypertension (82.7%), accompanied by high prevalence of heart disease (78.1%), dyslipidemia (69.3%), gastritis (68.9%), diabetes (43.9%), arthritis (90.4%), lung disease (44.3%), and stroke (24.1%). This class represented severe multisystem involvement [47] and was labeled the “Severe Cardiometabolic-Multisystem Pattern.”

Class 2 (22.3%) was characterized by extremely high probabilities of hypertension (99.7%) and arthritis (99.6%). Gastritis showed a moderate prevalence (37.5%), while dyslipidemia (27.3%) and heart disease (26.6%) were present in about one-quarter of individuals. Other conditions were rare (<13%), indicating a pattern primarily dominated by hypertension and joint disease. This class was labeled the “Hypertension-Arthritis Pattern.”

Class 3 (15.6%) was dominated by lung disease (90.8%), with co-occurrence of arthritis (58.7%), asthma (50.4%), gastritis (43.4%), and hypertension (35.3%). This class was labeled the “Respiratory-Musculoskeletal Pattern.”

Class 4 (24.1%) exhibited typical metabolic syndrome features, including high prevalence of hypertension (88.3%), dyslipidemia (54.5%), and heart disease (44.4%), with moderate diabetes (36.3%) and gastritis (33.4%). Other diseases were less common (<14%), indicating a primarily cardiometabolic profile without extensive multisystem involvement. This class was labeled the “Metabolic Syndrome Pattern.”

Class 5 (18.2%) had the lowest overall disease burden, dominated by arthritis (85.2%) and gastritis (76.4%), with all other diseases below 25%. This class was labeled the “Gastritis-Arthritis Pattern.” The naming of latent multimorbidity patterns was guided by the dominant disease combinations and their clinical interpretation, following conventions widely used in epidemiologic and multimorbidity-cluster studies [19,48-52].

### Performance Evaluation Results of CLA-Net

**Figure 5.**
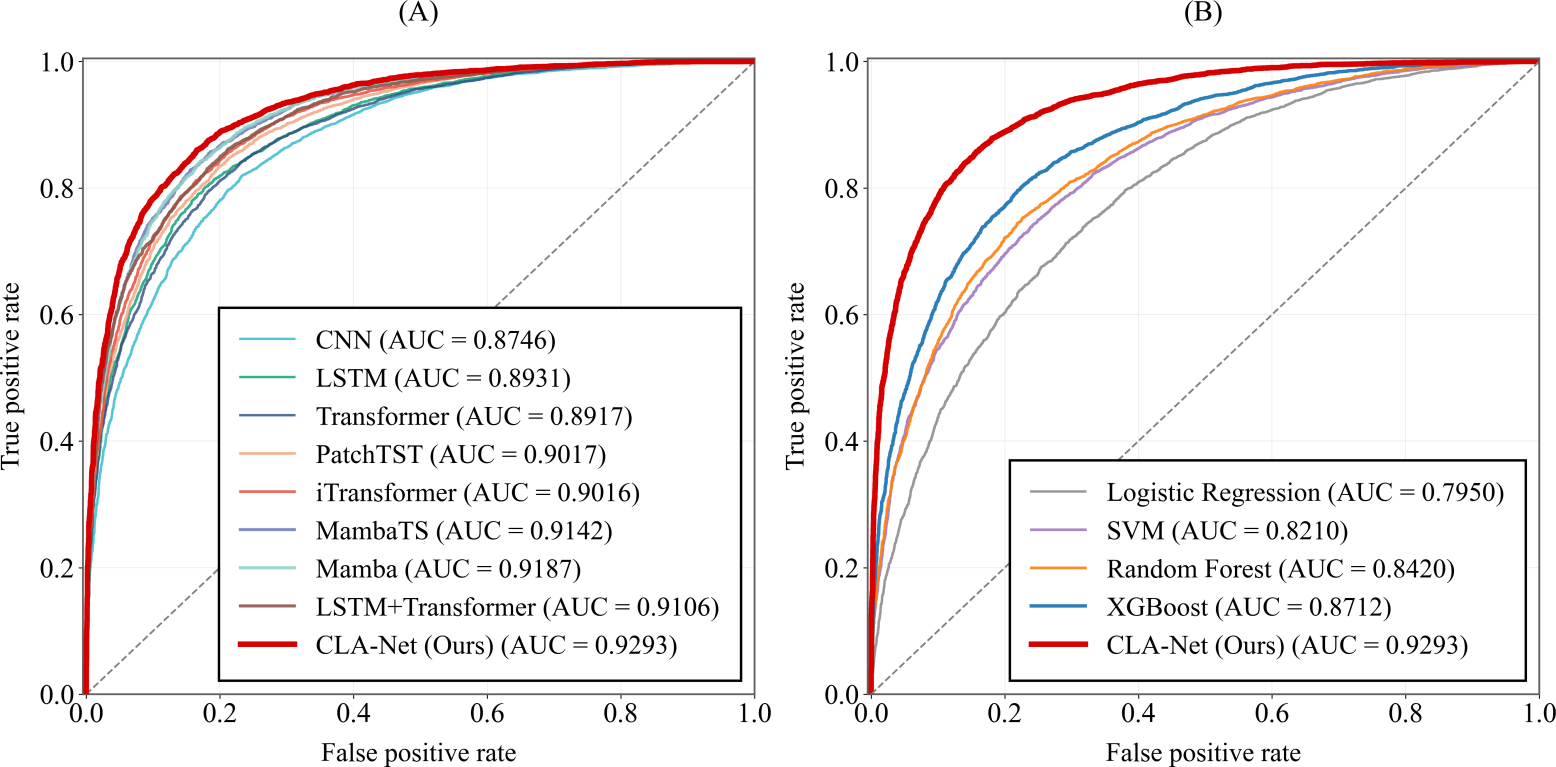
The area under the receiver operating characteristic curve of the Cross-Lag Attention Network and baseline models. AUC: area under the curve; CLA-Net: Cross-Lag Attention Network; CNN: convolutional neural network; LSTM: long short-term memory; SVM: support vector machine.

In the experiments, the proposed method was trained on the training set and evaluated on an independent test set. To ensure robustness, the entire process was repeated 50 times, and the mean and SD of all evaluation metrics across these runs were reported as the final performance results. The comparative performance of all models on the multimorbidity pattern prediction task is summarized in Table 5, the ROC curves are presented in Figures5  6, and the detailed results of the ablation study are shown in Table 6.

As shown in Table 5, CLA-Net consistently outperformed all baseline models in multimorbidity pattern prediction. Traditional machine learning approaches perform relatively poorly, while deep learning models designed for temporal modeling yield substantial improvements. Among the baselines, Mamba performed best overall, while convolutional neural network (CNN) performed the worst, underscoring the importance of temporal feature modeling in longitudinal health data. The hybrid LSTM+transformer model achieved relatively high accuracy, highlighting the benefits of combining sequential modeling with attention mechanisms. Nevertheless, CLA-Net surpassed all baselines across accuracy, precision, recall, and *F*_1_-score, with low SDs confirming its stability and robustness.

We further conducted formal statistical significance testing to assess whether the observed performance gains of CLA-Net over baseline models were statistically robust. Specifically, 2-sided Wilcoxon signed-rank tests were applied to paired performance scores obtained from 50 repeated experimental runs for each evaluation metric. To account for multiple comparisons across baseline models and metrics, *P* values were adjusted using the Holm step-down procedure. The Wilcoxon signed-rank test results are given in Table 7. The results indicate that CLA-Net achieves statistically significant performance improvements over all baseline models. The consistent significance across repeated experiments confirms that the observed gains are robust rather than driven by random fluctuations.

**Table 5.** The results of the performance comparison.^a^

Types of models and models	Precision	Recall	*F*_1_-score	Accuracy
Machine learning models, mean (SD)				
Logistic regression	0.7385 (0.0075)	0.7350 (0.0080)	0.7320 (0.0082)	0.7335 (0.0079)
SVM^b^	0.7540 (0.0069)	0.7510 (0.0074)	0.7480 (0.0076)	0.7495 (0.0072)
Random forest	0.7650 (0.0062)	0.7620 (0.0068)	0.7590 (0.0070)	0.7605 (0.0066)
XGBoost	0.7856 (0.0059)	0.7820 (0.0062)	0.7835 (0.0060)	0.7892 (0.0052)
Deep learning models, mean (SD)				
CNN^c^	0.7835 (0.0090)	0.7779 (0.0084)	0.7807 (0.0086)	0.7860 (0.0082)
LSTM^d^	0.8020 (0.0063)	0.8040 (0.0070)	0.8030 (0.0066)	0.8041 (0.0069)
Transformer	0.8138 (0.0079)	0.8074 (0.0076)	0.8106 (0.0072)	0.8120 (0.0069)
iTransformer	0.8167 (0.0074)	0.8189 (0.0078)	0.8178 (0.0071)	0.8184 (0.0070)
PatchTST	0.8092 (0.0082)	0.8057 (0.0088)	0.8074 (0.0081)	0.8088 (0.0083)
MambaTS	0.8191 (0.0061)	0.8214 (0.0067)	0.8202 (0.0062)	0.8210 (0.0064)
Mamba	0.8226 (0.0062)	0.8258 (0.0068)	0.8242 (0.0061)	0.8242 (0.0064)
LSTM + Transformer	0.8234 (0.0060)	0.8256 (0.0065)	0.8245 (0.0062)	0.8247 (0.0058)
CLA-Net (our model)	*0.8326 (0.0053)*	*0.8312 (0.0056)*	*0.8319 (0.0051)*	*0.8352 (0.0048)*

a The italicized values represent the best performance of each data handling strategy on the evaluation metrics. These italicized values are used to highlight the most outstanding results among the different strategies.

b SVM: support vector machine.

c CNN: convolutional neural network.

d LSTM: long short-term memory.

**Figure 6.**
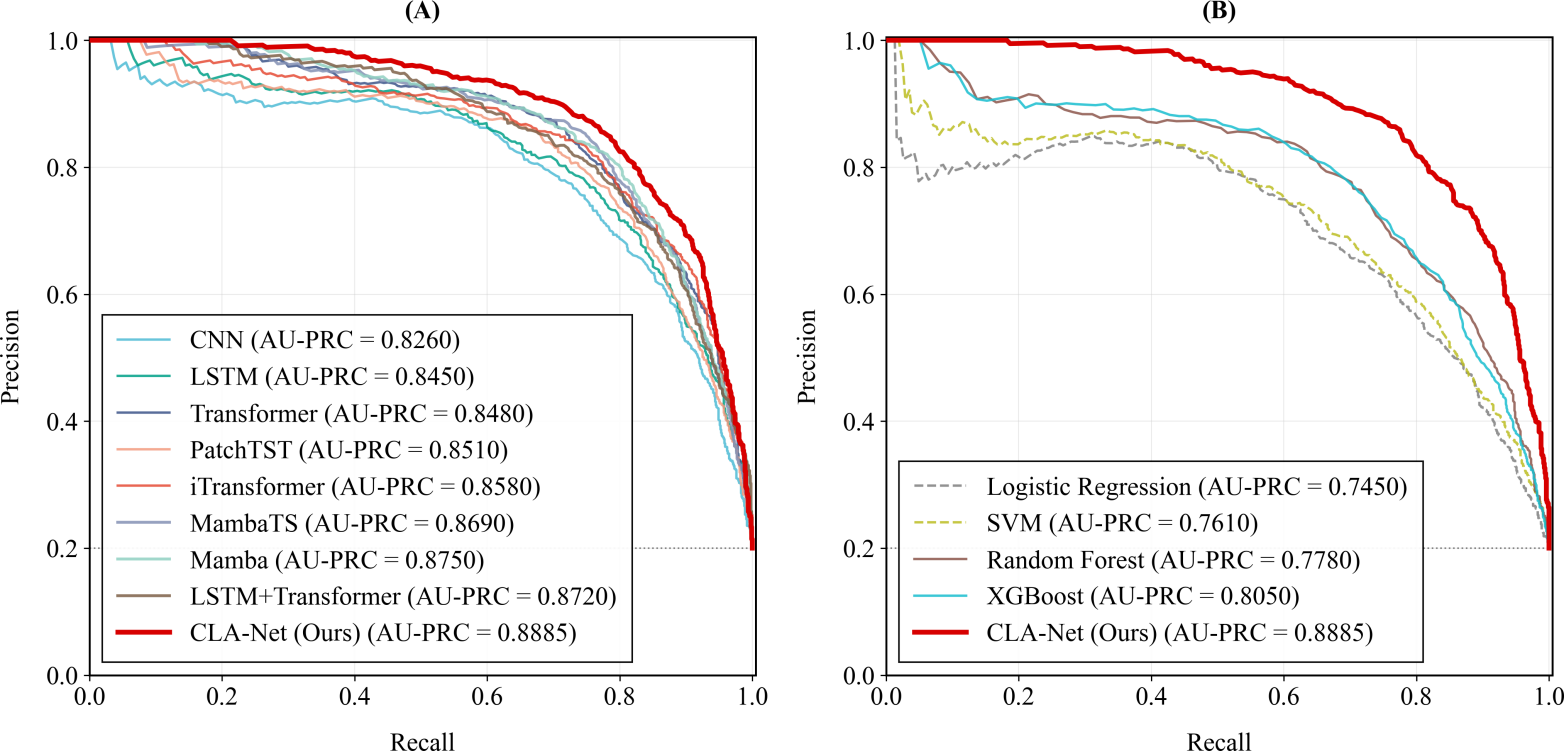
The area under the precision-recall curve of the Cross-Lag Attention Network and baseline models. AU -PRC: area under the precision-recall curve; CLA-Net: Cross-Lag Attention Network; CNN: convolutional neural network; LSTM: long short-term memory; SVM: support vector machine.

**Table 6.** Results of the ablation study.^a^

Type and ablation setup		Precision, mean (SD)	Recall, mean (SD)	*F*_1_-score, mean (SD)	Accuracy, mean (SD)
Full model					
	CLA-Net^b^ (our model)	*0.8326 (0.0053)*	*0.8312 (0.0056)*	*0.8319 (0.0051)*	*0.8352 (0.0048)*
Module ablation					
	Transformer branch	0.8153 (0.0068)	0.8116 (0.0071)	0.8134 (0.0069)	0.8166 (0.0065)
	GRU^c^ branch	0.8079 (0.0074)	0.8067 (0.0078)	0.8073 (0.0075)	0.8102 (0.0072)
	Replacing the bitemporal directed cross-attention mechanism with self-attention	0.8218 (0.0059)	0.8196 (0.0062)	0.8207 (0.0060)	0.8244 (0.0057)
Input configuration ablation					
	Single-time-point input	0.7982 (0.0083)	0.7964 (0.0087)	0.7973 (0.0085)	0.7985 (0.0082)
	Three-time-point input	*0.8338 (0.0055)*	0.8265 (0.0058)	0.8301 (0.0056)	0.8311 (0.0053)
	Four-time-point input	0.8251 (0.0057)	*0.8316 (0.0061)*	0.8283 (0.0059)	0.8287 (0.0056)
Architecture design					
	Reversing the order of transformer and GRU components	0.8230 (0.0061)	0.8208 (0.0064)	0.8219 (0.0062)	0.8235 (0.0059)

a The italicized values represent the best performance of each data handling strategy on the evaluation metrics.

b CLA-Net: Cross-Lag Attention Network.

c GRU: Gated Recurrent Unit.

**Table 7.** Statistical test results.^a^

Baseline	Precision *W*	Recall *W*	*F*_1_-score *W*	Accuracy *W*
Logistic regression	0***	0***	0***	0***
SVM^b^	0***	0***	0***	0***
Random forest	0***	0***	0***	0***
XGBoost	0***	0***	0***	0***
CNN^c^	0***	0***	0***	0***
LSTM^d^	0***	0***	0***	0***
Transformer	1***	0***	0***	1***
iTransformer	13***	68***	20***	15***
PatchTST	0***	0***	0***	0***
MambaTS	27***	82***	39***	0***
Mamba	52***	258***	101***	11***
LSTM+transformer	80***	227***	126***	11***

a Two-sided Wilcoxon signed-rank tests were conducted on paired metric scores obtained from 50 repeated runs (n=50) to compare Cross-Lag Attention Network against each baseline model; Reported W corresponds to the Wilcoxon test statistic; To control for multiple comparisons across 12 baselines and 4 metrics, *P* values were adjusted using the Holm procedure; ***: *P*<.001.

b SVM: support vector machine.

c CNN: convolutional neural network.

d LSTM: long short-term memory.

Figure 5 presents the AUC-ROC curves comparing CLA-Net with a range of baseline models. As shown in Figure 5A, among deep learning approaches, CLA-Net achieves the highest AUC of 0.9293, demonstrating superior discriminative ability in multimorbidity pattern prediction. Models explicitly designed for temporal modeling, such as Mamba (AUC=0.9187), MambaTS (AUC=0.9142), PatchTST (AUC=0.9017), and iTransformer (AUC=0.9016), outperform conventional CNN-based architectures, highlighting the importance of capturing long-range temporal dependencies in longitudinal health data. The hybrid LSTM+transformer model attains an AUC of 0.9106, ranking among the strongest deep learning baselines, while CNN yields the lowest AUC (0.8746) within this group.

To further assess performance under class imbalance, Figure 6 reports the PR curves and corresponding AUC-PRC values. As shown in Figure 6, CLA-Net achieves the highest AUC-PRC among deep learning models (0.8885), indicating a superior PR trade-off across recall levels. Temporal models such as Mamba, iTransformer, and LSTM+transformer also perform competitively, whereas CNN shows relatively weaker performance. Figure 6 demonstrates that CLA-Net substantially outperforms traditional machine learning baselines, including logistic regression, support vector machine, random forest, and XGBoost. Overall, the PRC results complement the ROC analysis and further confirm the robustness of CLA-Net in identifying future multimorbidity patterns under imbalanced conditions.

As summarized in Table 6, the full CLA-Net consistently outperformed all ablation variants, underscoring the importance of integrating both GRU and the bitemporal directed cross-attention mechanism. Removing either component or replacing cross-attention with self-attention led to clear performance drops, confirming the effectiveness of the original design.

Regarding input configurations, single-time-point input resulted in the largest decline, while 3-time-point and 4-time-point settings provided only marginal gains but reduced overall accuracy, indicating that the 2-time-point design achieves the optimal balance, efficiently capturing the most relevant temporal signals without introducing the complexity or noise associated with longer historical windows. These results indicate that, under the current architecture, incorporating immediate temporal context is critical for prediction, whereas extending the input window beyond 2 time points yields diminishing or even negative returns. In addition, reversing the order of transformer and GRU also impaired performance, supporting the strategy of “temporal encoding before interaction.”

**Figure 7.**
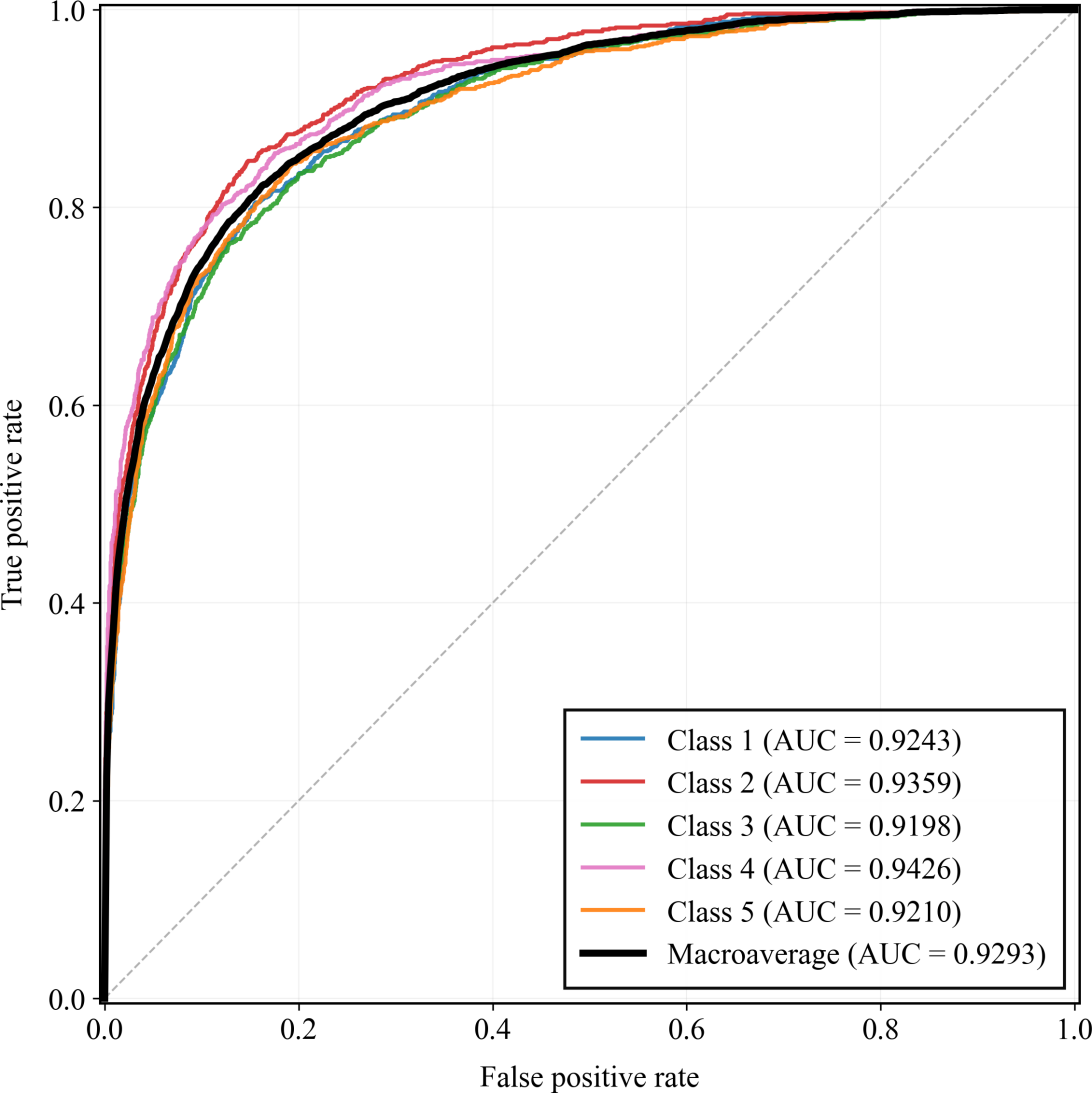
Multiclass area under the receiver operating characteristic curve of the Cross-Lag Attention Network. AUC: area under the curve.

Figures7  8 show the class-specific ROC and PR curves for the 5 multimorbidity patterns. All classes achieved consistently high ROC performance, with AUCs ranging from 0.9198 to 0.9426 and a macroaverage of 0.9293. The PRC analysis yielded AUC-PRC values between 0.8310 and 0.8620 (macroaverage=0.8429), indicating stable precision-recall trade-offs across classes. Overall, CLA-Net demonstrates robust and balanced predictive performance for all multimorbidity patterns.

To complement this threshold-free evaluation, Table 8 reports classwise precision, recall, and *F*_1_-scores, indicating consistently good predictive performance across patterns, with *F*_1_-scores ranging from 0.7906 to 0.8508. As further illustrated by the confusion matrix in Figure 9, classification errors are primarily concentrated among clinically related multimorbidity patterns, suggesting that residual misclassifications arise from intrinsic overlap between multimorbidity profiles rather than systematic model failure.

**Figure 8.**
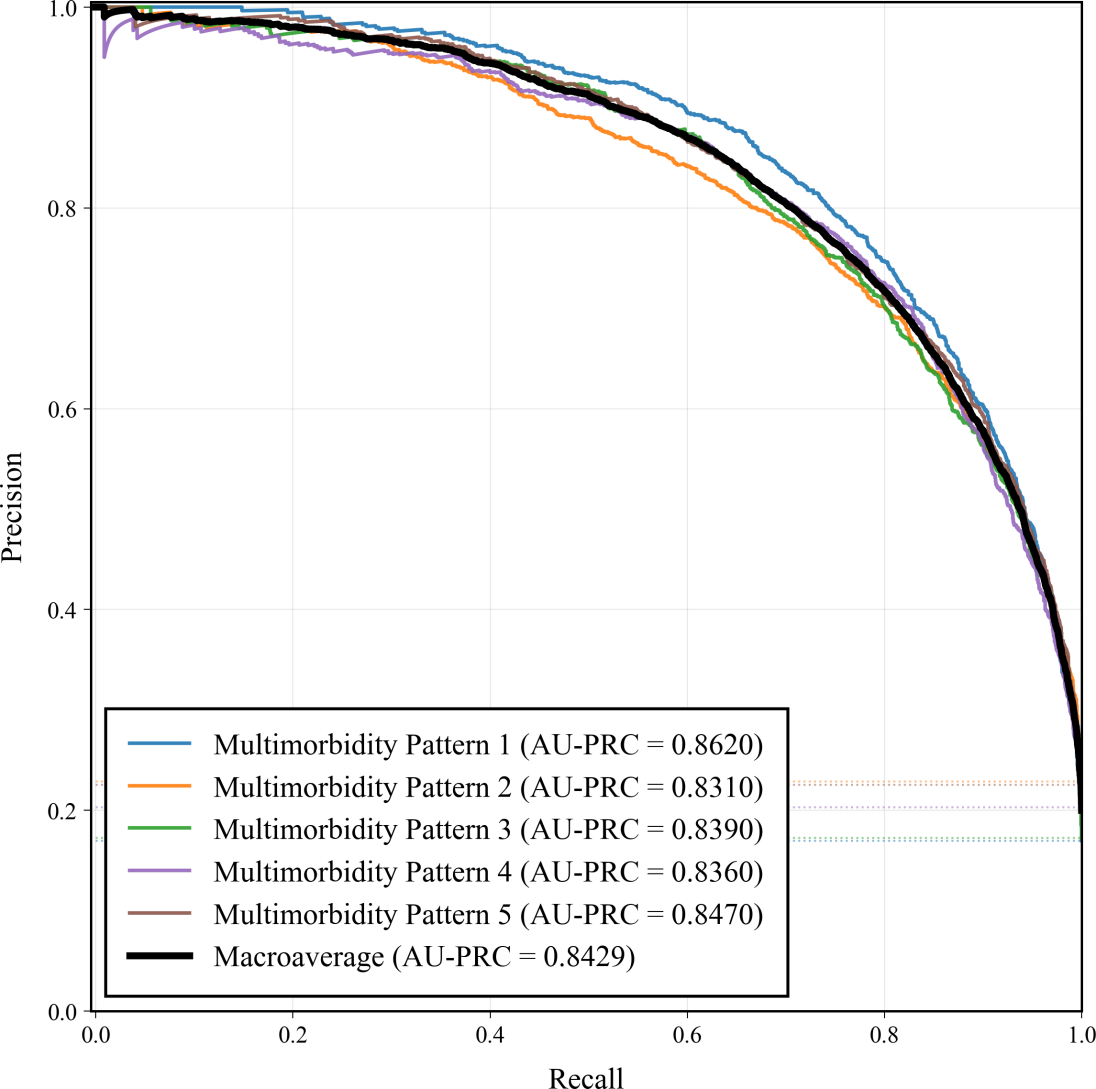
Multiclass area under the precision-recall curve of Cross-Lag Attention Network. AU-PRC: area under the precision-recall curve.

**Table 8.** Detailed classwise performance metrics of Cross-Lag Attention Network.

Multimorbidity pattern (class)	Prevalence (%)	Precision, mean (SD)	Recall, mean (SD)	*F*_1_-score, mean (SD)
C1: Severe Cardiometabolic-Multisystem	19.80	0.8403 (0.006)	0.8314 (0.007)	0.8359 (0.006)
C2: Hypertension-Arthritis	22.30	0.8402 (0.005)	0.8515 (0.006)	0.8458 (0.005)
C3: Respiratory-Musculoskeletal	15.60	0.8002 (0.009)	0.7813 (0.010)	0.7906 (0.009)
C4: Metabolic Syndrome	24.10	0.8480 (0.006)	0.8535 (0.006)	0.8508 (0.006)
C5: Gastritis-Arthritis	18.20	0.8221 (0.007)	0.8194 (0.008)	0.8207 (0.007)
Macroaverage	—^a^	0.8303 (0.007)	0.8274 (0.008)	0.8288 (0.007)
Weighted average (overall)	100	0.8326 (0.006)	0.8312 (0.007)	0.8319 (0.006)

a Not applicable.

**Figure 9.**
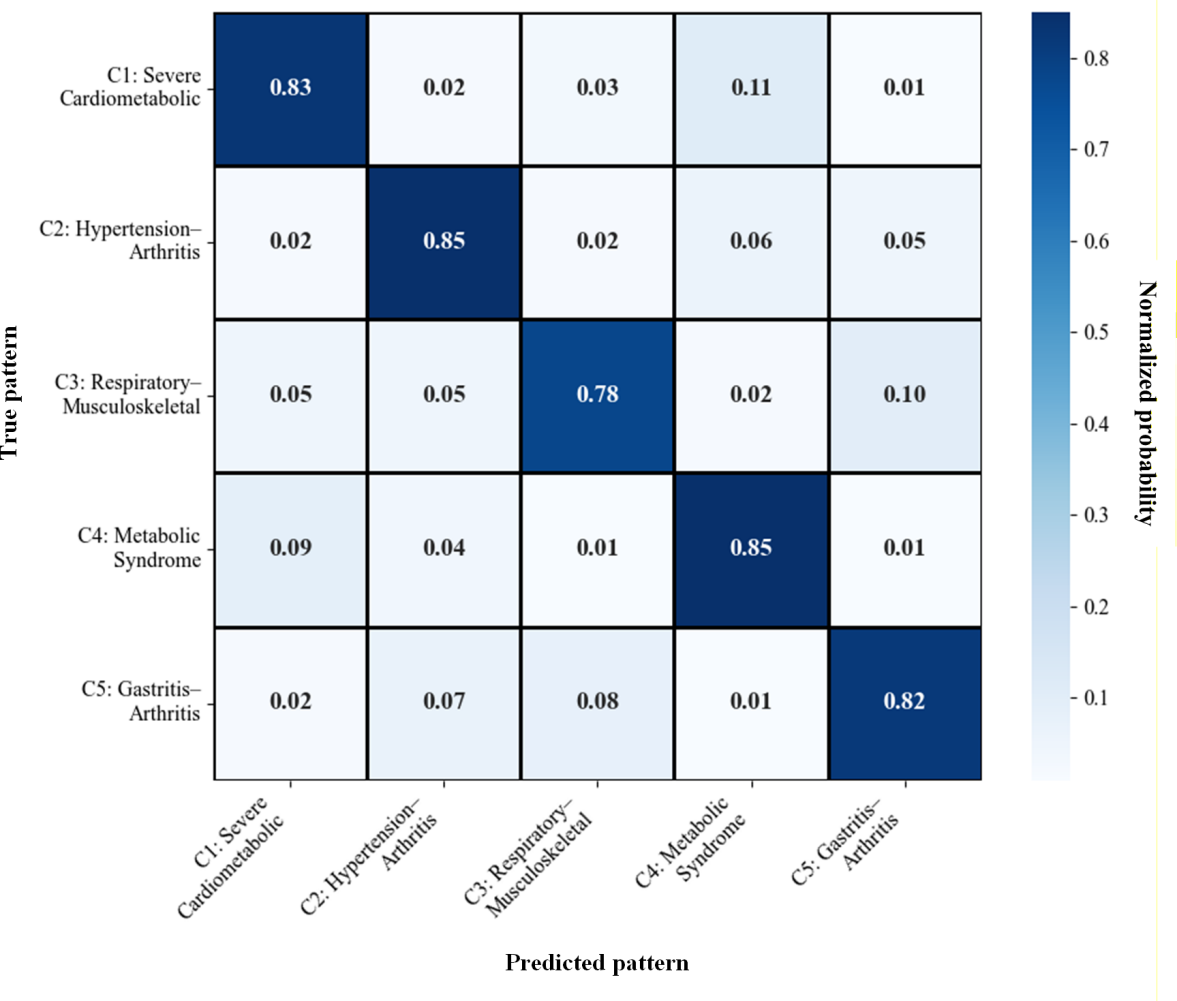
Confusion matrix across the 5 multimorbidity patterns.

### Sensitivity Analysis: Robustness of Class Stability

To address concerns regarding potential information leakage and the temporal robustness of latent class definitions, we conducted a comprehensive sensitivity analysis in which the LTA was reestimated using only the training-period waves (2011‐2015), thereby excluding all future observations used as prediction targets in the main analysis. This analysis was designed to evaluate whether the multimorbidity pattern structure identified in the full longitudinal dataset was inherently stable, rather than being driven by information from later waves.

We first reassessed the optimal number of latent classes by fitting LTA models with 2 to 6 classes under a free-parameter specification using the training-period data only. As shown in Table 9, information criteria (AIC, BIC, and SaBIC) consistently improved as the number of classes increased from 2 to 5, while a marginal deterioration in fit was observed when moving from 5 to 6 classes. The BLRT strongly supported the 5-class solution over the 4-class solution (*P*<.001), whereas the improvement from 5 to 6 classes was only marginal (*P*=.03), suggesting diminishing returns with increased model complexity.

**Table 9.** Model fit of latent transition analysis based on training-period waves (2011‐2015).

Models (waves used) and number of classes		AIC^a^	BIC^b^	SaBIC^c^	Entropy	BLRT^d^ (*P* value)
LTA^e^ (free parameters; 2011-2015)						
	2	6128.4	6316.2	6201.7	0.72	—^f^
	3	5486.9	5759.8	5594.3	0.8	<.001
	4	5142.6	5500.7	5284.9	0.84	<.001
	5	4926.8	5369.9	5104.2	0.87	<.001
	6	4954.1	5482.4	5166.5	0.85	.03
Selected model (2011-2015)						
	5	4926.8	5369.9	5104.2	0.87	—
LTA (invariant)						
	5	4968.3	5389.6	5136.8	0.86	—

a AIC: Akaike Information Criterion.

b BIC: Bayesian Information Criterion.

c SaBIC: sample-size adjusted Bayesian Information Criterion.

d BLRT: Bootstrap Likelihood Ratio Test.

e LTA: latent transition analysis.

f Not applicable.

In addition, classification quality, as reflected by entropy, reached its highest value under the 5-class solution (entropy=0.87), indicating satisfactory class separation despite the reduced number of waves and observations compared with the full-sample model. Taken together, these results demonstrate that the 5-class structure remained the optimal and most parsimonious representation of multimorbidity patterns even when the analysis was restricted to the training-period data.

To ensure consistency of class interpretation across time, measurement invariance constraints were imposed on the selected 5-class model. Although imposing invariance resulted in a modest increase in information criteria and a slight reduction in entropy (from 0.87 to 0.86), the overall classification quality remained high, indicating that the core latent structure was robust to parameter constraints and not dependent on future observations.

Beyond overall model fit, we directly examined the stability of class definitions by comparing disease-specific item-response probabilities between the full-sample LTA model and the training-period-only LTA model. For each latent class, the top 4 representative chronic conditions were retained, and their conditional probabilities were contrasted across the 2 modeling strategies (Table 10).

**Table 10.** Concordance of class definitions between full-sample latent transition analysis and training-only latent transition analysis.

Multimorbidity pattern (class) and top 4 representative conditions^a^	Probability (full model)^b^	Probability (training period only)^c^	Difference (bias)	Pearson correlation coefficient (*r*)^d^
Class 1: Cardiometabolic-Multisystem				0.954
Arthritis	0.904	0.774	−0.130	
Hypertension	0.827	0.854	0.027	
Heart disease	0.781	0.781	0.000	
Dyslipidemia	0.693	0.662	−0.031	
Class 2: Hypertension-Arthritis				0.983
Hypertension	0.997	0.917	−0.080	
Arthritis	0.996	0.958	−0.038	
Gastric disease	0.374	0.446	0.072	
Dyslipidemia	0.273	0.331	0.058	
Class 3: Respiratory-Musculoskeletal				0.970
Lung disease	0.908	0.783	−0.125	
Arthritis	0.587	0.604	0.017	
Asthma	0.504	0.540	0.036	
Gastric disease	0.434	0.466	0.032	
Class 4: Metabolic Syndrome				0.973
Hypertension	0.883	0.841	−0.042	
Dyslipidemia	0.545	0.646	0.101	
Heart disease	0.444	0.346	−0.098	
Diabetes	0.363	0.308	−0.055	
Class 5: Gastritis-Arthritis				0.965
Arthritis	0.852	0.892	0.040	
Gastric disease	0.764	0.818	0.054	
Heart disease	0.242	0.302	0.060	
Kidney disease	0.194	0.253	0.059	
Overall stability				
Average across all classes	—^g^	—	0.048^e^	0.969^f^

a To ensure consistency, the top 4 conditions with the highest prevalence in the full model were selected for comparison for each pattern.

b Derived from the complete dataset, including Waves 1 through 5 (2011‐2020).

c Derived exclusively from the training dataset (Waves 1 through 3, 2011‐2015), blinding the model to future test data.

d Pearson correlation coefficient calculated based on the probability vectors of all 14 chronic conditions for the corresponding class.

e Mean absolute deviation value.

f Mean value.

g Not applicable.

Across all 5 classes, the composition and relative ordering of dominant conditions remained highly consistent. The cardiometabolic-multisystem class continued to be characterized by a high prevalence of arthritis, hypertension, heart disease, and dyslipidemia; the hypertension-arthritis class preserved its defining dual dominance of hypertension and arthritis; the respiratory-musculoskeletal class remained anchored by lung disease and asthma; the metabolic syndrome class retained its core metabolic features; and the gastritis-arthritis class continued to reflect co-occurring gastrointestinal and musculoskeletal conditions. Importantly, no class exhibited a change in its defining disease constellation when future waves were excluded from model estimation.

Quantitatively, the similarity between item-response probability profiles was high across all classes, with Pearson correlation coefficients ranging from 0.954 to 0.983 and a mean correlation of 0.969. These correlations were computed using the full set of disease-specific probabilities, indicating strong structural concordance rather than superficial agreement limited to a subset of conditions [53]. Differences in absolute probabilities were generally modest, with a mean absolute deviation of 0.048 across classes, reflecting only minor shifts in disease prevalence rather than substantive changes in class meaning.

Taken together, these findings provide strong evidence that the multimorbidity class structure identified in the main analysis is stable and not driven by information from future observations. Even when latent classes were defined exclusively using training-period data, both the optimal number of classes and their clinical interpretations remained highly consistent with those derived from the full-sample model. The observed differences were limited to modest shifts in absolute disease probabilities, while the underlying pattern structure and relative disease importance were preserved.

We acknowledge that, under a strictly unbiased evaluation framework, latent class definitions could alternatively be derived exclusively from training-period data and then applied to the test set, for example, by projecting test observations onto a training-derived latent space. In this study, however, we adopted a population-level LTA fitted on all available waves to define stable multimorbidity patterns, which were subsequently treated as fixed targets for prediction. Importantly, as demonstrated in Table 9, the latent class structures derived from training-period data and from the full dataset exhibited a high degree of concordance (mean *r*=0.969), indicating that the class definitions were not driven by any single wave or subset of the data. Therefore, the use of the full-sample LTA model to generate predictive targets does not introduce meaningful bias into the evaluation. The “ground truth” labels for the test set would remain virtually unchanged even if they were generated by projecting test data onto a latent space fitted strictly to training parameters. The reported predictive performance of CLA-Net reflects a genuine ability to forecast future multimorbidity states rather than an artifact of information leakage in the target generation process.

To explicitly assess whether the use of full-sample LTA targets introduces any optimistic bias in predictive evaluation, we conducted an additional experiment in which the LTA model was estimated exclusively on training-period data, with all model parameters fixed thereafter. The downstream prediction pipeline was then fully repeated using these training-only LTA-derived targets, and the CLA-Net model was retrained and reevaluated accordingly.

As summarized in Table 11, the predictive performance obtained under training-only LTA targets remains highly consistent with the original results based on full-sample LTA targets. Specifically, the differences in accuracy, precision, recall, and *F*_1_-score are all below 0.3%, and all metrics remain well within 1 SD of the original estimates. The slight performance reductions observed are expected and can be attributed to minor numerical variations in latent class assignment, rather than to any systematic bias arising from information leakage.

**Table 11.** Comparison of predictive performance under full-sample and training-only LTA^a^ targets.

Metric	Full-sample LTA targets, mean (SD)	Training-only LTA targets, mean (SD)	Difference
Accuracy	0.8352 (0.0048)	0.8338 (0.0050)	−0.0014 (−0.17%)
Precision	0.8326 (0.0053)	0.8315 (0.0055)	−0.0011 (−0.13%)
Recall	0.8312 (0.0056)	0.8290 (0.0060)	−0.0022 (−0.26%)
*F*_1_-score	0.8319 (0.0051)	0.8302 (0.0054)	−0.0017 (−0.20%)

a LTA: latent transition analysis.

### Transition-Focused Evaluation of Multimorbidity Class Prediction

To disentangle the prediction of genuine multimorbidity progression from trivial class persistence, we conducted a stratified evaluation by separating individuals into those whose multimorbidity class remained unchanged between consecutive waves (stayer-only) and those who experienced a true class transition (transition-only). Performance was assessed using macroaveraged precision, recall, *F*_1_-score, and overall accuracy for each subset.

To further account for chance agreement and the high prevalence of class persistence, we additionally report chance-adjusted performance metrics. Across the full sample, CLA-Net achieved a Cohen κ of 0.7935 (SD 0.0055) and a Matthews correlation coefficient of 0.7968 (SD 0.0058), reflecting substantial chance-adjusted concordance between predicted and observed class labels. These values are consistent with the maintained performance on the transition-only subset, confirming that CLA-Net possesses robust discriminative power to identify genuine multimorbidity evolution rather than merely replicating static baselines.

As shown in Table 12, the model retained substantial discriminative ability on the transition-only subset, where individuals experienced a true change in multimorbidity class between tand t+1. On this subset, macroaveraged precision, recall, and *F*_1_-score reached 0.7615 (SD 0.0106), 0.7632 (SD 0.0112), and 0.7623 (SD 0.0102), respectively, with an overall accuracy of 0.7654 (SD 0.0096). Although performance on the transition-only subset was lower than that observed in the full-sample and stayer-only evaluations, this reduction is expected given the increased complexity of predicting off-diagonal class transitions. Because multimorbidity patterns tend to remain relatively stable across adjacent waves in populations with largely irreversible chronic conditions, the transition-only subset represents a smaller and more heterogeneous group, which inherently increases prediction difficulty and performance variability, as reflected by the larger SDs observed.

**Table 12.** Performance evaluation on different dynamic subsets.

Evaluation subset	Precision, mean (SD)	Recall, mean (SD)	*F*_1_-score, mean (SD)	Accuracy, mean (SD)
Full sample	0.8326 (0.0053)	0.8312 (0.0056)	0.8319 (0.0051)	0.8352 (0.0048)
Transition-only (*Y_t_* ≠ *Y_t_*_+1_; 23.47%)	0.7615 (0.0106)	0.7632 (0.0112)	0.7623 (0.0102)	0.7654 (0.0096)
Stayer-only (*Y*_*t*_ = *Y_t_*_+1_; 76.53%)	0.8544 (0.0049)	0.8521 (0.0052)	0.8533 (0.0047)	0.8566 (0.0044)

As an important clinical benchmark, we further evaluated a naive persistence model that simply predicts class(*t+1*)=class(*t*). On the test set, the naive model obtained an overall accuracy of 0.7653, substantially below CLA-Net (0.8352, SD 0.0048). Notably, on the transition-only subset, the naive model achieves 0% accuracy by definition, whereas CLA-Net maintains 0.7654 accuracy, demonstrating its added value in identifying genuine transitions rather than merely reproducing stable states. Together, these results indicate that the overall performance gain is not driven by trivial persistence, but by the model’s capacity to capture progression-related signals associated with changes in multimorbidity structure.

To further account for chance agreement and the high prevalence of class persistence, we additionally report chance-adjusted performance metrics. Across the full sample, CLA-Net achieved a Cohen κ of 0.7935 (SD 0.0055) and a Matthews correlation coefficient of 0.7968 (SD 0.0058), reflecting substantial chance-adjusted concordance between predicted and observed class labels. These values are consistent with the maintained performance on the transition-only subset, confirming that CLA-Net possesses robust discriminative power to identify genuine multimorbidity evolution rather than merely replicating static baselines.

### Sensitivity Analysis of Data Imputation Methods

To strictly verify the validity and robustness of the imputation strategy used in this study (ie, MiceForest), we conducted a comparative experiment to assess how different missing data handling methods affect the final predictive performance of CLA-Net. In this experiment, the standard MiceForest algorithm was replaced by 4 alternative strategies: complete case analysis (CCA; directly discarding samples with missing values), random imputation (filling missing values with randomly selected observed values), mean imputation (filling with the variable mean), and KNN imputation. The comparative results are presented in Table 13.

**Table 13.** Performance comparison of Cross-Lag Attention Network using different missing data handling strategies.^a^

Imputation strategy	Precision, mean (SD)	Recall, mean (SD)	*F*_1_-score, mean (SD)	Accuracy, mean (SD)
Complete case analysis	0.8015 (0.0072)	0.7980 (0.0078)	0.7995 (0.0075)	0.8050 (0.0070)
Random imputation	0.8080 (0.0085)	0.8050 (0.0090)	0.8065 (0.0088)	0.8110 (0.0082)
Mean imputation	0.8150 (0.0065)	0.8120 (0.0069)	0.8135 (0.0066)	0.8180 (0.0062)
KNN^b^ imputation	0.8265 (0.0058)	0.8240 (0.0062)	0.8252 (0.0060)	0.8290 (0.0055)
MiceForest (ours)	*0.8326 (0.0053)*	*0.8312 (0.0056)*	*0.8319 (0.0051)*	*0.8352 (0.0048)*

a The italicized values represent the best performance of each data handling strategy on the evaluation metrics. These italicized values are used to highlight the most outstanding results among the different strategies.

b KNN: k-nearest neighbors.

As shown in Table 13, the model using MiceForest for data imputation achieved the best performance across all metrics (accuracy=0.8352, *F*_1_-score=0.8319). In contrast, CCA resulted in the lowest performance (accuracy=0.8050). It is important to note that the CCA metrics were evaluated on a reduced test set (subset of individuals with complete data only), whereas imputation methods were evaluated on the full test set. Despite being tested on this potentially “cleaner” subset, CCA still underperformed. This confirms that the substantial reduction in training sample size caused by discarding data severely compromises the model’s ability to learn robust patterns, thereby limiting its generalizability. Random imputation also performed poorly (accuracy=0.8110), primarily because randomly assigned values introduce significant noise and disrupt the true correlation structure between variables. While mean imputation (accuracy=0.8180) and KNN imputation (accuracy=0.8290) showed improvements over random methods, they still lagged behind MiceForest.

These results indicate that MiceForest, which leverages iterative random forests to model non-linear interactions, effectively preserves the underlying data structure better than stochastic (random) or distance-based (KNN) methods, confirming it as the optimal choice for our framework.

## Discussion

### Principal Results

This study proposes an innovative research framework that integrates population-level multimorbidity pattern recognition with individual-level future prediction, achieving a key transition in multimorbidity research from descriptive statistics to prospective prediction.

In terms of multimorbidity pattern recognition, this study identified 5 clinically meaningful classes using LTA: Cardiometabolic-Multisystem, Hypertension-Arthritis, Respiratory-Musculoskeletal, Metabolic Syndrome, and Gastritis-Arthritis. Patients in the Cardiometabolic-Multisystem pattern bore the heaviest overall disease burden, with widespread hypertension, heart disease, dyslipidemia, diabetes, gastritis, arthritis, lung disease, and kidney disease, while the coexistence of stroke further indicated advanced vascular damage. The interactions and cascading effects among these chronic conditions have been confirmed in multiple studies. For instance, cardiometabolic diseases such as hypertension, diabetes, dyslipidemia, and heart disease frequently co-occur and significantly increase the risk of cardiovascular events and mortality [50]. Inflammatory arthritis (eg, rheumatoid arthritis) has also been associated with elevated cardiovascular risk [54]. This multisystem involvement highlights the importance of comprehensive and integrated management for such patients. The Hypertension-Arthritis pattern was characterized by the strong co-occurrence of hypertension and arthritis. This pattern is well supported by prior studies showing that hypertension is a common multimorbidity of osteoarthritis, potentially linked through shared mechanisms such as inflammation, oxidative stress, and vascular dysfunction, with arthritis treatments possibly influencing blood pressure [51,55]. The Respiratory-Musculoskeletal pattern was defined by a high prevalence of lung disease, arthritis, and asthma, often accompanied by gastritis and hypertension. This finding is consistent with previous clinical evidence [52,56]. The Metabolic Syndrome class exhibited high rates of hypertension, dyslipidemia, heart disease, and diabetes, without significant multisystem involvement. This aligns with the classical definition of metabolic syndrome and its strong association with increased risks of cardiovascular disease and type 2 diabetes [57]. Finally, the Gastritis-Arthritis pattern showed the lowest overall disease burden, primarily involving arthritis and gastritis. This finding is supported by prior medical studies reporting comorbidities such as enteropathic arthritis in patients with inflammatory bowel disease or gastrointestinal complications related to arthritis medications [58].

In multimorbidity pattern prediction, this study introduces CLA-Net, a novel deep learning framework specifically designed to forecast multimorbidity patterns from longitudinal health data with short temporal lags. Our comprehensive evaluation demonstrated that CLA-Net consistently outperformed all baseline methods, achieving an accuracy of 0.8352 and an AUC of 0.9293. Several strong deep learning baselines included in the comparison, such as Mamba, PatchTST, and iTransformer, were originally developed for long-sequence time series forecasting and are optimized to exploit long-range temporal dependencies through mechanisms such as selective state-space modeling or patch-based representations. In the present task, the input sequence consists of 2 adjacent time points *t−1* and *t*, which may limit the extent to which these architectures can fully leverage their design strengths. In contrast, CLA-Net explicitly focuses on modeling short-lag transitions through a dual-branch architecture and a bitemporal directed cross-attention mechanism, enabling more effective capture of immediate disease-state dependencies. Compared with the best-performing baseline Mamba model, CLA-Net improved accuracy by 1.10% (0.8352 vs 0.8242) and by 1.05% over the hybrid LSTM+transformer architecture (0.8352 vs 0.8247). More notably, CLA-Net increased AUC by 1.87% relative to LSTM+transformer (0.9293 vs 0.9106), indicating superior discriminative ability across decision thresholds, which is particularly important for clinical applications requiring flexible risk stratification strategies.

Our ablation experiments revealed the critical contributions of individual components and validated the soundness of the architectural design. Only the transformer branch reduced accuracy to 0.8166 (−1.86%), highlighting its essential role in capturing global cross-temporal dependencies. Retaining only the GRU branch further decreased accuracy to 0.8102 (−2.50%), indicating that while GRU effectively models temporal dynamics, the absence of global feature interactions severely limits its ability to capture complex multimorbidity patterns. The performance gap between the 2 branches (0.8166 vs 0.8102) also suggests that global interactions are slightly more important than local temporal dependencies, yet their integration is necessary to achieve optimal performance. Replacing the bitemporal directed cross-attention with standard self-attention reduced accuracy to 0.8244 (−0.93%), with precision and *F*_1_- scores declining by 1.08% and 1.12%, respectively. This underscores the importance of the directed mechanism in maintaining predictive stability and balance. Unlike standard self-attention, which may cause “future information leakage,” the dual-window design enforces a “history-to-current” information flow that better aligns with the clinical logic of predicting future risk based on historical data.

In terms of input configuration, single-time-point input resulted in a sharp drop in accuracy to 0.7985 (−3.67%) and recall to 0.7964 (−3.48%), indicating that the absence of historical information causes the model to miss many true multimorbidity cases, likely because early signals of certain patterns can only be detected through temporal evolution. Interestingly, adding more historical time points (3 or 4) did not improve overall performance: with 3 time points, precision slightly increased (0.8355 vs 0.8334) but accuracy dropped to 0.8228 and recall to 0.8185; with 4 time points, recall reached its highest (0.8360), but accuracy (0.8268) and *F*_1_-score (0.8262) fell below the 2-time-point configuration. This empirical finding suggests that for this specific multimorbidity prediction task, immediate history is significantly more predictive than long-term history. Including more distant waves (eg, from 6‐8 years prior) appears to introduce noise or weaker relevance that obscures the strong signals from the most recent health state transition. Therefore, the 2-time-point configuration effectively captures the critical “immediate” evolution while minimizing interference from outdated information, thus striking the best balance between information richness and model complexity. Importantly, this temporal design also has clear implications for practical clinical use. By leveraging a “past–current” window spanning approximately 2‐3 years to predict multimorbidity patterns 2‐3 years into the future, CLA-Net is not intended for short-term clinical decision-making (eg, acute care), but rather for midterm risk stratification and early intervention planning. In real-world settings, such predictions could be used to identify individuals who are likely to transition into more complex or higher-burden multimorbidity patterns within the next few years, thereby providing a clinically meaningful window for intervention. Finally, reversing the order of GRU and transformer (GRU-transformer) lowered accuracy to 0.8235 (−1.17%), confirming the effectiveness of the “temporal encoding before interaction” design. GRU provides temporally aware representations that serve as more suitable inputs for subsequent attention mechanisms, enabling more effective cross-temporal interactions, whereas applying attention without temporal context may lead to suboptimal feature modeling.

Considering the chronic and partially irreversible nature of many included conditions, class persistence is expected to be high. Consistent with this, approximately 76.53% of test instances were stayers. While the naive model achieves high accuracy on stable patients, it has zero clinical utility for risk warning, as it fails to identify any patient whose health state is deteriorating or changing (0% accuracy on the transition subset). In contrast, CLA-Net successfully identifies 76.54% of the patients who undergo pattern transitions. From a clinical perspective, the primary value of a predictive model lies in its ability to provide early warnings for high-risk transitions rather than merely confirming stability. Therefore, the complexity of the deep learning approach is justified by its ability to capture these critical, nonlinear disease progressions that a simple persistence rule completely misses.

Regarding generalizability, the proposed framework is designed to be transferable to other longitudinal electronic health record (EHR) datasets. The LTA component relies on routinely collected chronic disease diagnoses, which are widely available across EHR systems, allowing population-level pattern structures to be reestimated or adapted to different health care contexts. Meanwhile, the CLA-Net architecture operates on generic temporal feature representations and does not depend on dataset-specific coding schemes. This modular design facilitates scalability to larger cohorts and longer observation periods and enables potential transfer learning scenarios in which a pretrained representation model can be fine-tuned using locally derived multimorbidity structures. These properties suggest that the framework has the potential for broader application beyond the present dataset. However, due to the inherent survivorship bias of longitudinal follow-up cohorts, the current model is primarily applicable to chronic disease management in surviving, community-dwelling populations. Importantly, it should not be applied to high-acuity clinical settings (eg, emergency or Intensive Care Unit populations) where mortality risk is a competing outcome and may dominate near-term trajectories. In such contexts, mortality-aware endpoints and competing-risk modeling would be required prior to any clinical deployment.

### Practical Implications

The ability of CLA-Net to anticipate future multimorbidity pattern membership offers important implications for the longitudinal management of patients with established chronic conditions. Rather than responding to isolated disease events, clinicians can leverage pattern-based forecasts to organize care around expected configurations of coexisting conditions, thereby supporting a more coordinated and forward-looking approach to chronic disease management.

First, knowledge of an individual’s likely future multimorbidity pattern can inform proactive care planning within patients with multimorbidity. For example, individuals predicted to enter the metabolic syndrome pattern may benefit from earlier emphasis on lifestyle modification, metabolic regulation, and adherence support, with the aim of slowing progression toward more complex cardiometabolic profiles. Similarly, those expected to belong to the cardiometabolic-multisystem pattern can be prioritized for closer surveillance and integrated management across cardiovascular, metabolic, and musculoskeletal domains, helping to mitigate cumulative disease burden over time.

Second, pattern-level prediction provides a practical framework for structuring multidisciplinary collaboration. Instead of relying on uniform or reactive referral pathways, health care providers may tailor multidisciplinary team composition according to the anticipated constellation of conditions. For instance, the respiratory-musculoskeletal pattern underscores the value of coordinated input from pulmonology, rheumatology, and rehabilitation services to jointly address respiratory impairment and functional limitation. Likewise, the gastritis-arthritis pattern highlights the need for alignment between gastroenterology and pain management to balance long-term anti-inflammatory therapy with gastrointestinal protection.

At the organizational level, pattern-based predictions can facilitate more efficient allocation of follow-up intensity, monitoring priorities, and supportive services for patients with multimorbidity within long-term chronic care systems. Consistent with its design scope, the proposed framework does not seek to model acute deterioration, end-stage progression, or mortality-related outcomes. Rather, it functions as a scalable decision-support layer to identify patients who may benefit from anticipatory, pattern-oriented management strategies in settings focused on sustained multimorbidity care.

### Limitations and Future Direction

Although this study has made significant progress in multimorbidity pattern prediction, several areas remain for improvement. First, although CLA-Net demonstrated strong predictive performance on a longitudinal cohort, the current evaluation was conducted using a single data source. Differences in population characteristics, disease coding systems, and follow-up structures across EHR datasets may affect model behavior and warrant further investigation. Future work will focus on validating the framework across diverse EHR datasets, exploring transfer learning strategies, and extending the model to other chronic disease domains and health care systems.

Second, this study does not explicitly incorporate medication-related information into the modeling framework. Chronic conditions were therefore represented as binary indicators of presence or absence, without distinguishing between actively treated and untreated disease states. This abstraction is appropriate for predicting future multimorbidity pattern membership at the population level, but it may obscure clinically relevant differences in disease control achieved through pharmacological management. Consequently, the predicted pattern assignments should be interpreted as reflecting disease co-occurrence status rather than treatment-adjusted clinical responses. Future work integrating longitudinal medication data may further enhance the clinical granularity of multimorbidity pattern prediction.

Third, the model’s predictive performance reflects a combined but functionally distinct reliance on objective disease history and subjective self-assessment measures, such as self-rated health and self-rated memory. While objective disease variables anchor predictions to diagnosed multimorbidity status, subjective assessments provide higher-level summaries of perceived health and functional burden and may contribute more strongly for individuals with borderline or heterogeneous disease profiles. Although the proposed framework demonstrates strong overall predictive performance, the relative contributions of subjective versus objective inputs cannot be fully disentangled within the current framework without dedicated interpretability analyses, such as feature attribution or attention visualization. Addressing this limitation and providing greater transparency regarding the sources of predictive power will be a key focus of future work.

Fourth, although the proposed framework demonstrates strong predictive performance, incorporating detailed interpretability analyses, such as SHAP (Shapley Additive Explanations) values or attention weight visualizations, may further enhance its practical utility. Owing to the complexity of the model architecture, such analyses are beyond the scope of this study. Future research will focus on developing tailored interpretability strategies to better elucidate the learned multimorbidity representations and support clinical interpretation.

Fifth, regarding temporal granularity, the survey waves in the CHARLS dataset are separated by nonuniform intervals, ranging from approximately 2 to 3 years. In the current CLA-Net architecture, transitions between adjacent waves are modeled as equidistant steps, without explicitly encoding the varying time intervals. This simplification may introduce temporal noise into the estimation of transition dynamics, particularly when interpreting changes across waves with heterogeneous follow-up durations. Future work could address this limitation by incorporating time-aware representations or modeling strategies explicitly designed for irregular temporal intervals.

Another methodological consideration concerns the transformation of probabilistic latent class assignments into deterministic labels for supervised prediction. In this study, posterior class probabilities obtained from the LTA were converted into hard labels using a maximum posterior probability assignment strategy. While this approach provides a clear and stable supervisory signal for training the prediction model, it inevitably discards information regarding classification uncertainty. In particular, individuals with ambiguous or borderline posterior distributions are treated as belonging entirely to a single class, which may encourage overconfident predictions. Future work could address this limitation by leveraging soft targets, such as full posterior probability vectors, within the learning objective. Incorporating probabilistic supervision through distribution-based loss functions or uncertainty-aware architectures may enable a more nuanced representation of multimorbidity structure and further improve predictive robustness.

Finally, to ensure the integrity of longitudinal trajectory modeling and the stability of LTA patterns, this study included participants present in all 5 waves (2011‐2020). We acknowledge that excluding individuals who died or dropped out due to severe illness may introduce survivorship bias, potentially skewing the dataset toward a “healthier survivor” cohort. However, this inclusion criterion was a necessary methodological trade-off to accurately capture the continuous evolution of multimorbidity patterns. It is worth noting that the primary goal of this framework is to support chronic disease management and secondary prevention in the general population, rather than predicting acute mortality risk in end-stage patients. To address this limitation, future research could incorporate advanced strategies such as joint longitudinal-survival modeling, inverse probability-of-censoring weighting, or competing-risk frameworks to explicitly account for nonrandom attrition and mortality, thereby extending the model’s generalizability to high-risk clinical populations.

### Conclusion

This study addresses the complex challenges of multimorbidity management in the context of population aging by proposing an innovative framework that bridges population-level pattern recognition and individual risk prediction. Using LTA, we identified multimorbidity patterns with temporal consistency and clinical stability (ie, Cardiometabolic-Multisystem, Hypertension-Arthritis, Respiratory-Musculoskeletal, Metabolic Syndrome, and Gastritis-Arthritis), which were then transformed into predictive labels to develop the CLA-Net deep learning model. CLA-Net integrates the sequential modeling capacity of GRU with the feature interaction advantages of a bitemporal directed cross-attention mechanism, achieving superior performance in capturing both temporal dependencies and complex feature interactions in chronic disease progression. Experimental results demonstrated that CLA-Net significantly outperformed existing methods across accuracy, recall, precision, *F*_1_-score, and AUC.

## Supplementary material

10.2196/84261 Multimedia Appendix 1 Final dataset variables.

## Abbreviations

AIC: Akaike Information Criterion
AU-PRC: area under the precision-recall curve
AUC: area under the curve
BIC: Bayesian Information Criterion
BLRT: Bootstrap Likelihood Ratio Test
CCA: complete case analysis
CHARLS: China Health and Retirement Longitudinal Study
CLA-Net: Cross-Lag Attention Network
CNN: convolutional neural network
EHR: electronic health record
FFN: feed-forward network
FPR: false positive rate
GRU: Gated Recurrent Unit
KNN: k-nearest neighbors
LCA: latent class analysis
LSTM: long short-term memory
LTA: latent transition analysis
PR: precision-recall
ROC: receiver operating characteristic
SaBIc: sample-size adjusted Bayesian Information Criterion
SHAP: Shapley Additive Explanations
TPR: true positive rate
