# Supplementary material for: Bridging Population Patterns and Individual Prediction: Framework for Prospective Multimorbidity Study
Source: JMIR Med Inform. 2026 Mar 10;14:e84261. doi: 10.2196/84261 (PMC12983216; doi:10.2196/84261)
Supplement: Multimedia Appendix 1 [file medinform-v14-e84261-s001.docx]

Table S1. Included variables.

| Categories | Variables | Data type | Range |
| --- | --- | --- | --- |
| Baseline characteristics | ID | String | / |
|  | Year | String | 2011, 2013, 2015, 2018, 2020 |
| Chronic diseases | Hypertension | **Boolean** | Yes/No |
|  | Dyslipidemia | **Boolean** | Yes/No |
|  | Diabetes | **Boolean** | Yes/No |
|  | Cancer | **Boolean** | Yes/No |
|  | Lung diseases | **Boolean** | Yes/No |
|  | Liver diseases | **Boolean** | Yes/No |
|  | Heart diseases | **Boolean** | Yes/No |
|  | Stroke | **Boolean** | Yes/No |
|  | Kidney diseases | **Boolean** | Yes/No |
|  | Gastric disease | **Boolean** | Yes/No |
|  | Mental disorder | **Boolean** | Yes/No |
|  | Memory disorder | **Boolean** | Yes/No |
|  | Arthritis | **Boolean** | Yes/No |
|  | Asthma | **Boolean** | Yes/No |
| Symptoms | Arthritis | **Boolean** | Yes/No |
|  | Asthma | **Boolean** | Yes/No |
|  | Headache | **Boolean** | Yes/No |
|  | Shoulder pain | **Boolean** | Yes/No |
|  | Arm pain | **Boolean** | Yes/No |
|  | Wrist pain | **Boolean** | Yes/No |
|  | Finger pain | **Boolean** | Yes/No |
|  | Chest pain | **Boolean** | Yes/No |
|  | Stomach pain | **Boolean** | Yes/No |
|  | Back pain | **Boolean** | Yes/No |
|  | Waist pain | **Boolean** | Yes/No |
|  | Hip pain | **Boolean** | Yes/No |
|  | Leg pain | **Boolean** | Yes/No |
|  | Knee pain | **Boolean** | Yes/No |
|  | Ankle pain | **Boolean** | Yes/No |
|  | Toe pain | **Boolean** | Yes/No |
|  | Neck pain | **Boolean** | Yes/No |
| Other Individual Characteristics | Gender | Category | Male/Female |
|  | Age | Numeric | / |
|  | BMI | Category | Underweight / Normal weight / Overweight / Obese |
|  | Waist-to-Height Ratio (WHtR) | Numeric | / |
|  | Nighttime sleep | Numeric | / |
|  | Nap duration | Numeric | / |
|  | Exercise | Boolean | Yes/No |
|  | Drinking | Boolean | Yes/No |
|  | Smoking | Boolean | Yes/No |
|  | Life satisfaction | Category | Not at all satisfied/Slightly satisfied/Moderately satisfied/Very satisfied/Extremely satisfied |
|  | Cognitive ability | Numeric | 0–21 points |
|  | Mental health problems | Numeric | 0–30 points |
|  | Marital status | Boolean | Yes/No |
|  | Coresidence with children | Boolean | Yes/No |
|  | Weekly communication with children | Boolean | Yes/No |
|  | Weekly communication with parents | Boolean | Yes/No |
|  | Monthly social engagement | Boolean | Yes/No |
|  | Child satisfaction | Category | Very Dissatisfied/ Dissatisfied/ Neutral / Neither satisfied nor dissatisfied/ Satisfied/ Very Satisfied |
|  | Financial aid received from children (past year) | Boolean | Yes/No |
|  | Financial aid received from parents (past year) | Boolean | Yes/No |
|  | Educational level | Category | Less than junior high school/High school and vocational training  /Higher education |
|  | Urban/rural residence | Category | Urban/Rural |
|  | Per Capita household consumption | Numeric | / |
|  | Total household income | Numeric | / |
|  | Pension insurance | Boolean | Yes/No |
|  | Childhood adversity index | Numeric | 0–9 points |
|  | Social isolation | Numeric | 0–4 points |
|  | Hobbies | Boolean | Yes/No |
|  | Household size | Numeric | / |
|  | Self-rated memory | Numeric | 0–4 points |
|  | Medical insurance | Boolean | Yes/No |
|  | Work or not | Boolean | Yes/No |
|  | Retire or not | Boolean | Yes/No |
|  | Self-rated health | Numeric | 0–4 points |
|  | Hospitalization in the past year | Boolean | Yes/No |
|  | Experience of major traffic accidents | Boolean | Yes/No |
